# Report on the Progress of Human Anatomy and Physiology in the Years 1844-5

**Published:** 1846-07

**Authors:** James Paget

**Affiliations:** Lecturer on General and Morbid Anatomy and Physiology, and Warden of the Collegiate Establishment, at St. Bartholomew's Hospital


### PART FOURTH.

### Original Reports and Memoirs.

### REPORT ON THE PROGRESS OF HUMAN ANATOMY AND PHYSIOLOGY IN THE YEARS 1844-5.

### PART II.

By James Paget,

Lecturer on General and Morbid Anatomy and Physiology, and Warden of the College, at St. Bartholomew's Hospital.

### NUTRITION.

*In its Chemical Relations.—Formation of Fat.* I cannot find, in the long course of experimental pig-fattening described by M. Boussingault,* anything that is both new and interesting to human physiology. He and M. Persoz† who has fattened geese, do not appear to have found out more than was already known, namely, that the presence of fatty matter in the food is not essential to the formation of fat in the body; and that a certain quantity of nitrogenous principles in the food is essential to that end; the mere truth being that for an animal to grow soundly fat it must be in tolerable health, and that for this it must have some of its natural diet.

In relation to the well-proved formation of fat from the saccharine principles of the food, an interesting observation is made by H. Meckel.‡ He finds that when grape-sugar and bile are mingled fatty matter is formed from the former; thus, in his chief experiment, performed under the guidance of Marchand, 440 grammes of ox-bile were divided into four parts: the first, while recent, was treated with ether; the second was so treated after exposure for twenty-four hours to the heat of an incubating machine; to each of the third and fourth portions, there were added 4 grammes of grape-sugar obtained from starch; and the third was exposed for five hours, and the fourth for twenty-four hours, to the heat of the incubating machine. Then, from these third and fourth portions, all the fatty matter was extracted by ether. The quantities of fatty matter thus obtained were, from the first portion, ·48 grammes; from the second, ·54; from the third, ·87; from the fourth,

* Annuaire de Chimie, 1846, p. 789, from the Ann. de Chimie et de Physique, t. xiv.

† Report from the Acad. des Sciences, 16 Decembre, 1844, in the Gazette Médic., 21 Decembre. Three of the principal papers on the formation of fat, by MM. Edwards, Boussingault, and Persoz, which have been noticed in the last two Reports, are fully reprinted in the Annales de Chimie et de Physique, Août, 1845. They are all admirably criticised by Liebig, in the Annalen der Chemie und Pharmacie, Juni 1845; and in the Lancet, of the same month.

‡ De Genesi Adipis in Animalibus; Halis, 1845, 8vo. I did not obtain this treatise in time to mention the fact as a contribution to the physiology of the liver.

1·84. The much larger quantity of fatty matter in the third and fourth portions can be assigned only to the sugar which was added to the bile; and the greater quantity in the fourth than in the third appears to show the gradual progress of the transformation, which was not completed in the last portion even after twenty-four hours; for sugar unchanged could still be detected in it.

It is thus made probable that the process of transformation of the amylaceous principles of the food into fat consists in their being, first, by the saliva and pancreatic-fluid, transformed into grape-sugar, of which some is converted by the bile into fatty matter in the intestines, and the rest, absorbed by the vessels leading to the portal vein, is carried in its branches to the liver, and therein is also converted into fatty matter. This is confirmed by an experiment of Trommer, who, having fed animals on grape-sugar, detected it in the blood of the portal vein, but not in that of the hepatic veins. Neither is it impossible that a similar series of transformations should be effected in carnivorous animals; the gelatine of their food being, perhaps, converted into sugar of gelatine, and this into fatty matter.

*Quantity of Nitrogen in Food.* Drs. Schlossberger and Kemp* have constructed a table of what they suppose to be the comparative proportions of nutriment in our several organic aliments; but it is scarcely more than a table of the comparative proportions of nitrogen in them. It is too long to be extracted, and cannot be analysed.

*Proportions of Elements discharged in the Excretions.* Some experiments on chickens, by Dr. Sace,† show the respective quantities of the elements of their food which are discharged by the cloacal excrements, and by transpiration. Two chickens consumed in a week, in barley, chalk, and sand, 211·544 grammes of carbon, 30·1551 of hydrogen, 10·6123 of nitrogen, 197·468 of oxygen, and 15·4695 of substances which would have remained as ash after combustion; and their cloacal excrements yielded of carbon 50·3946 grammes, of hydrogen 6·7275, of nitrogen 4·3524, of oxygen 45·9836, and of inorganic matter remaining as ash 121·6128. Their joint increase in weight was 19·18 grammes; allowing for this, the quantities of the several constituents which were transpired and retained in the tissues may be easily reckoned.

### NUTRITION IN ITS RELATIONS TO STRUCTURE.

*Theory of Cell-Development.* A very lucid exposition of this theory, and of the principal facts concerning the history and nature of the nucleated cell in the structures of animals, has been published by Kölliker.‡ The subject has also been thoroughly discussed by Reichert§ in his 'Report on the Progress of Microscopic Anatomy in 1843,' his observations being included in an examination of essays by Karsten,‖ Kölliker,¶ and Nägeli.** The general tendency of the whole is to show that we are yet very far from the knowledge of the true mode of development of the nucleated cell in animals. There is indeed in all these essays, as well as in the personal knowledge of most anatomists, an abundance of facts bearing upon the subject; but many, perhaps

* Lond. and Edinb. Philos. Mag., Nov. 1845; Medical Gazette, Dec. 12, 1845; and Annalen der Chemie, Oct. 1845.

† Annalen der Chemie, Oct. 1844. He seems to think his experiments show what proportion of food is assimilated, and what is at once excreted without being first assimilated; but they do not do this.

‡ In Schleiden and Nägeli's Zeitschrift, Heft ii, 1845; another part is announced for publication, but I have not been able to obtain it.

§ In Müller's Archiv, 1844, No. vi. Jahresbericht, pp. 148-172.

‖ De Cella Vitali; Berol. 1843. See last Report.

¶ Entwickelungsgeschichte der Cephalopoden; Zurich, 1844.

** Zur Entwickelungsgeschichte des Pollens; Zurich, 1842; and in Schleiden and Nägeli's Zeitschrift für Botanik, 1844, Heft i.

the majority, of these facts cannot be brought within the expressions of Schwann's theory of cell-development; neither can there be yet traced in them any single, uniform, and constant mode of development of the nucleated cell. From the very nature of the case, it seems most probable that one law and one mode must be always observed in the development of the cell and its parts; if it be so, the one mode is unknown; if it be not so, then, in the place of the fair and comprehensive system of Schwann we have a crowd of unconnected facts such as no memory can contain, and of which it would be useless, even if it were possible, to give a brief report.

The observations of Reichert, as well as those in the other works just referred to, relate only, or principally, to the genesis of the nucleated cell and its several parts; and he implies that there is much less room for doubt concerning the metamorphoses of the cell itself, by which, of it or through it, all the more highly organized animal tissues are supposed to be formed. It appears to me, however, that we can be as little sure of many of the changes which nucleated cells are said to pass through in the formation of other tissues, as we are of the process by which the cells themselves are formed. The development of all the fibrous tissues appears especially doubtful. For the investigations of every year show the great difficulty or impossibility of confirming the observations by which Schwann explained the development of these and some other tissues, and the equal facility of finding appearances which cannot be reconciled with his theory, or any other single theory yet proposed concerning it.*

I have found ample reason for expressing these doubts of the sufficiency of the accepted theories of development in recent examinations of tumours and other morbid growths. Their structure seems peculiarly adapted for testing a theory of cell-development; for they are, doubtless, obedient to the same general laws of formation as the healthy structures are, and, in the unequal and often rapid growth of their several parts, it could hardly happen but that in many specimens all the phases would be seen through which their structures pass towards their fully developed state. But in very numerous examinations I have not found a single example in which a cell has appeared to be forming or formed around a pre-existing nucleus; or one in which fibres have appeared to be formed out of nucleated cells; or one in which nucleated cells have appeared to constitute a stage towards any form of higher development. On the contrary, I have found many instances of rapidly growing structures composed of large collections of fibres without a nucleated cell among or near them; others with abundant nucleated cells, but scarcely any free nuclei or granules, and nothing like a cell incompletely developed round its nucleus; and, again, others (and these of especially rapid growth) with no cells at all, but composed almost entirely of corpuscles like nuclei or cytoblasts.

From these and other observations I am disposed to think that the ordinary (and not the exceptional) mode of development of fibres is, not through nucleated cells, but from a structureless or dimly granular substance which is first *marked*, and then broken up, into fibres. There is good evidence that the cytoblasts which are usually or always imbedded in this substance, influence the development of the fibres; and though I cannot tell how they do so, yet it is certainly not by conversion of themselves into fibres; they shrivel and disappear as the fibres increase and become more perfectly formed.

I think it will be found that, in morbid growths, the nucleated cell is always a terminal, not a transitional, form; for in certain tumours in which the best formed nucleated cells are found, e. g. the epithelial tumours and some

* A good evidence of this is in the fact that the most original observers, when they speak of the development of the tissues, almost always cease for the time to be original, and copy both the words and drawings of Schwann or Valentin.

examples of medullary cancers, there are no higher forms found, not even imperfect fibro-cellular tissue, except in small quantity about the blood-vessels. Corpuscles having the characters of nuclei or cytoblasts (to adopt still the usual names) appear to be the really energetic bodies in the growth and determination of these morbid structures; they are found in some tumours so abundantly, and so unmixed with nucleated cells, that their power of multiplying and assimilating cannot be doubted; and it is in some of these tumours also that, apparently under the influence of the cytoblasts, the most perfect fibro-cellular tissue is ultimately formed. What I have seen also of the development of these cytoblasts, leads me to agree with that view of the development of nuclei generally, according to which they are described as formed, not on a pre-existent nucleolus, but out of granules collected in a dark or dim mass of the proper size and shape, which then clears up by the formation of a membranous wall and transparent fluid contents with, in some cases, one or more persistent granules holding the position of nucleoli.

*Anatomy of Nucleated Cells.* Although, if the doubts just expressed are well founded, we may have lost the thread for weaving the facts concerning cells into a system, still they must be collected with peculiar interest; for they must at some time be the basis of structural physiology.

In the last Report, several observations were mentioned concerning the molecular movements of particles within cells. Additions have been made during the year to the most interesting of these, namely those, such as Dr. Sharpey first observed, in which the movements are regular and in currents analogous to the currents of particles in the chara and other vegetables.

Professor Czermak* has described peculiar rotatory movements of particles in certain vesicles attached to the fine extremities of the seminal tubes of the black salamander. The vesicles are either attached to the peritoneal folds connecting the seminal tubes, or are imbedded in the tubes themselves; and the rotatory movements begin as soon as water is brought into contact with them. The vesicles are spherical or oval, from one fiftieth to one seventieth of an inch in diameter, and are covered by an outer layer of polygonal, mutually-flattened, granular cells. They contain, 1. round, oval, or pear-shaped corpuscles, some of which are not unlike blood-corpuscles, and which vary much in size, but have an average diameter of 1-2000th of an inch. 2. In many of the larger vesicles there is one large corpuscle, not granular, and occupying from one tenth to two thirds of the cavity, the rest of which contains corpuscles like those last described, or crystals. 3. The largest vesicles contain crystals of uncertain nature, alone or with the corpuscles before mentioned.

The rotatory movements vary according to the contents of the cells. The smaller corpuscles (of the first kind) move as if ciliæ within the vesicles impelled them. In some they move from side to side; in others, round their axes; in some, up and down; in some oval vesicles they move along the middle, from one end to the other, backwards and forwards. The movements usually continue for several hours, are accelerated by warmth, and are stopped by drying, or by completely soaking the vesicles in water. The vesicles which contain the larger (second kind of) corpuscles, show a different movement of their contents No ciliæ could be discerned on the large corpuscles, yet the small ones move along their margins just as if ciliæ seated there impelled them; and the large corpuscles themselves move slowly round their own axes in one constant direction.

The nature and mode of generation of the vesicles is quite uncertain; they at first were taken for the ova of entozoa, but the author gives reasons for thinking that they are not, and holds them to be analogous to those which Remak† found in the mesogastrium of frogs and has described under the name of ciliary vesicles.

* Oesterr. Medic. Jahrbucher, Jan. 1845.

† Müller's Archiv, 1841, H. v.

Kolliker* also has observed movements analogous to these within cells in two lower animals, namely, in the cells of the seminal filaments of *Polyclinum stellatum,* and in large cells in the sprouting arms of a young medusa-like radiate animal.

*Centres of Nutrition.* Mr. Goodsir† believes that the theory which he holds‡ of the existence of "germinal spots" in the secreting glands, i. e. of "a number of points from which acini are developed as from so many centres," may be extended to the process of development and nutrition of all the organs and textures. In an obscure exposition of his theory, he calls the points whose office corresponds to that of the germinal spots in glands, the "centres of nutrition," of the several textures. Each of these centres he considers to be "a cell, the nucleus of which is the permanent source of successive broods of young cells which from time to time fill the cavity of their parent, and then carrying with them the cell-wall of the parent, pass off in certain directions and under various forms, according to the texture or organ of which the parent forms a part." He names that a "germinal membrane," in which "the nutritive or germinal centres are arranged at equal or variable distances, and in certain directions in the substance of a fine transparent membrane." Such a membrane, identical with that which Mr. Bowman has named the basement-membrane, forms the tubules of glands, and the secreting epithelium is situated on its inner surface ; its nuclei are the germinal spots, or centres of nutrition. "A germinal membrane is occasionally found to break up into portions of equal size, each of which contains one of the germinal centres ;" showing that it "consists of cells with their cavities flattened, so that their walls cohering at their edges form the membrane, and their nuclei remain in its substance as the germinal centres." The secondary cells developed from the germinal spots or nutritive centres of such a membrane are always attached on its free surface ; they are at first contained between the two layers of the membrane, (these two layers being formed by the opposite walls of its component cells,) and when fully developed they carry forward the superficial layer, leaving the nuclei or germinal centres in the substance of the posterior or deeper layer in contact with the blood-vessels.

The theory is illustrated by the example of serous membranes.§ Their germinal membrane is the layer immediately below the epithelium. It does not, in general, show the lines of jnnction of its component flattened cells. These, its cells, appear to be elongated in the form of ribands ; their nuclei, or the germinal spots, being also elongated, expanded at one end, elongated at the other, somewhat bent, and directed, in general, parallel to the subjacent blood-vessels, in the neighbourhood of which they are most numerous. These flattened riband-shaped scales or cells, and the bright crystalline nuclei, appear identical with Henle's nucleus-fibres. (In aged and inflamed serous membranes, they appear to break up into areolar tissue). It is assumed that the nuclei are the sources of all the [epithelium] scales of the superficial layer of the serous membrane, each being the source of those in a certain compartment of its own ; and that, in the development of these epithelium-scales, the necessary nutritive material passes from the blood in the adjacent capillaries to the several centres, from each of which the scales of a compartment derive their origin and their nourishment, till they are detached.

Again, another example is referred to in the bones ; || in which, as already stated, the mass of soft cells in each bone-corpuscle is considered to be the nutritive centre or germinal spot for all the cells within the range of the canaliculi of that corpuscle.

* Entwick. der Cephalopoden, p. 156 ; in Reichert's Jahresbericht, u. s. p. 171 ; and in Schleiden u. Nägeli's Zeitschr. u. s, p. 101.

† Anatomical and Pathological Researches ; Edinb. 1845, p. 1.

‡ L. c. p. 29, and Trans. Roy. Soc. of Edinburgh, 1842. § L. c. p. 41. || L. c. p. 65.

*Development of Tissues.* Mr. Owen* thus describes the development of dentine. The cells at the base of the dentine-pulp fall into linear series, directed towards the periphery of the pulp; while those at and close by the periphery, being already similarly arranged in series, become more closely aggregated and enlarged, and change as follows. The large granular nucleus of each increases, becomes more opaque around a pellucid point in its centre, and then divides through its long axis. The division is succeeded by a further transverse subdivision of each nucleus; and the subdivisions of each become elongated, with their long axes vertical to the plane of the pulp, and then attach themselves to the corresponding elongated and subdivided nuclei of cells in advance. The attached extremities of the nuclei become confluent, and form a linear, or rather, a wavy, series of granular matter.

While these changes are going on, the calcareous salts begin to be deposited—1st, they accumulate within the cells; 2d, they are aggregated in a semitransparent state round the confluent elongated granular nuclei, which now appear as secondary cells; and 3d, they occupy, in a still clearer and more compact state, the interspaces of the cells. The nuclear tracts, i. e. the series of granular nuclei, receiving a smaller proportion of the salts than the other parts do, constitute the areæ of the dentinal tubes; the nucleolar membranes, or secondary cells, become the proper walls of the dentinal tubes; the indications of the proper walls of the parent-cells are retained through a modification of the arrangement of the calcareous salts in and between them; and the salts deposited within the parent-cells and around the secondary cells convert the intermediate spaces into the intertubular substance. The primary curves of the dentinal tubes depend on the primary linear series of the parent-cells; the secondary curves are determined by the angles at which the subdivided and elongated nuclei unite when their extremities coalesce in the wavy series already mentioned. As the calcification proceeds in its centripetal course, the pulp decreasing in size, fewer nuclei are developed in the cells, and these are smaller. Thus it happens that the linear tract formed by the nuclear matter in a smaller cell unites with the converging extremities of two tracts of a cell in advance. Thus the bifurcation of a tube is produced; and the repetition of this, which becomes more frequent as the calcifying process approaches the base of the pulp, gives rise to the dichotomous division of the main tubes.

The tissues which Mr. Owen has named osteo-dentine and vaso-dentine, acquire their peculiar structure from the change which take place in the central smaller cells of the pulp being different from those just described. In the formation of osteo-dentine the cell retains its nucleus undivided, and the salts are imparted around it within the cell, but enter only partially into its granular substance. And in the formation of vaso-dentine many of the cells lose their nuclei, which appear to be dissolved. In both these substances the blood-vessels of the pulp remain; in true dentine they wholly disappear.

Mr. Owen's account of the development of enamel agrees with that generally received. He suggests that the transverse striæ of the human enamel-fibre may be caused by the remains of multiplied nucleoli subdividing or modifying the walls of the elongated enamel-cells.

In the development of the cement, the blastema of the capsule acquires a fine granular structure, in which the calcareous salts are imparted in a comparatively clear state, constituting the framework of the cemental tissue. They penetrate the cavities of the nucleated cells, which are arranged in concentric rows around the part already calcified and which rest in cup-shaped cavities in its periphery; but their progress is arrested by the large granular nuclei, which maintains an irregular area, partly occupied by the salts in a subgranular

* Odontography, p. xliii-lxi. The account given by Mr. Tomes, in his excellent Lectures on Dental Physiology, now in course of publication in the 'Medical Gazette,' is in all its principal parts nearly similar to Mr. Owen's.

opaque condition. From these nuclear cavities are subsequently developed the minute radiated tubes (calcigerous canals). But these cavities are not formed in the layer of cement which covers the crown of the human and some other simple teeth; the layer of capsule in which it is developed contains no nucleated cells.

*Growth of Parts.* A series of micrometric observations has been made by Professor Harting,* with the view, chiefly, of determining the changes of number and dimension which the elements of each tissue and organ undergo in their development and growth from the early period of fœtal life to adult age. His results show that the elements of the tissues may be thus placed in two classes: 1st, those elementary structures which, from their first existence to adult life, increase in size either very little or not at all; so that the growth of the tissue which they form must be ascribed to their multiplication, not their enlargement; and 2d, those which constantly increase in size, till the tissue or organ which they form has gained its full dimensions, while their number does not increase after birth; the growth of the whole organ depending on their enlargement, not on their multiplication. In the first class he places the cells of epithelia, the fibrils of fibro-cellular tissue and tendons, the primitive fibrils of voluntary muscles, the cellular cavities of bones, and the blood-corpuscles. In the second class are the cells of the black choroidal pigment, of the fat, and of the liver, the primitive fasciculi (fibres) of voluntary muscles, and the fibres of involuntary muscle, the primitive nerve-tubules, the cells of cartilage, the urine-tubes and Malpighian capsules; and, probably, the fibres of elastic tissue and the ganglion-corpuscles. In a third class he says we might place a few elementary structures the number of which appears even to decrease after birth, e. g. the primitive fibres of muscles and the cells of permanent cartilage.

### STRUCTURE AND FUNCTIONS OF THE URINARY ORGANS.

The *Structure of the Kidney* has been studied by Drs. Gerlach,† Bidder,‡ and Kölliker,§ who all confirm the description of Mr. Bowman in nearly every particular—only they all find that the Malpighian body or tuft of vessels does not lie naked or bare within the capsule. The first two describe it as covered by a layer of epithelium, reflected on it from the walls of the capsule, like the reflected or visceral layer of a serous membrane; and they admit a space existing between this reflected layer of epithelium and that by which the capsule is lined. Kölliker thinks there is no such space, but that the Malpighian body is imbedded in one continuous layer of epithelium, which on the one side covers and fits into all the spaces between its vessels, and on the other is attached to the structureless membrane which forms the wall of the capsule.

Gerlach also says that the Malpighian capsules (which he has examined in the injected kidneys of sheep) are not at the ends of the tubules, but are attached like diverticula to their sides—[but they are certainly terminal in the human kidney; and Bidder and Kölliker assert that they are so in the kidneys of the frog and the triton: they may be lateral in the sheep; but it is more probable that they only appear so when two tubules lead to one capsule. Whenever this happens, as it does sometimes in other than sheep's kidneys, the capsule which is really at the junction of the ends of two tubes may appear like a lateral diverticulum on one]. Gerlach, moreover, holds (as Valentin does) that the ciliary epithelium in the frog's kidney extends over the whole internal surface of the capsule; but neither Kölliker nor Bidder agrees to this.

Kölliker's observations were made in connexion with others on the primordial kidneys or Wolffian bodies, the structure of which he has found to be

* Recherches micrométriques, 4to; Utrecht, 1845.

‡ Ibid. Heft v, p. 508.

‡ Ibid. Heft v, p. 518.

almost identical with that of the kidneys. They consist of tortuous tubules, which are formed by structureless membrane, lined by three or more layers of laminated epithelium-cells, the innermost layer of which bears long ciliæ. The ends of these tubules are dilated into Malpighian capsules, which are lined by a more delicate epithelium without ciliæ, and in each of which there is a Malpighian body, a tuft of capillary vessels, entering and leaving at the part opposite the connexion of the capsule with the tubule, and covered by a layer of cells.*

*Excretion of Urine.* In a boy affected with extroversion of the bladder, Mr. Erichsen† has observed the mode in which the urine flows through the ureters, and the rapidity with which substances pass from the stomach through the blood into the urine. His observations on the mode of excretion agree with others already made. When a drop of urine has collected within the termination of the ureter, which is elevated on a small papilla, the orifice of the canal opens to a diameter of two or three lines, and then, when the drop has passed it, contracts with a sphincter-like action. When the patient has neither eaten nor drunk for twelve hours, about three drops of urine pass every minute through each ureter, but at no regular intervals; neither is the action of the two ureters simultaneous, or regularly alternate. When the patient lies down, the urine does not flow at all for some time, and then flows slowly and gently, with a less distinct opening and shutting of the orifice of the ureter. Then, if he stands erect, after having been some time recumbent, it flows in a full stream till the ureters have emptied themselves of what had collected in them. During deep inspiration or straining, the flow of urine is suddenly increased, and it escapes in a small stream or in several rapidly succeeding large drops. Its excretion is made twice as rapid by violent exercise as it is during rest, and is peculiarly influenced during digestion. For fifteen or twenty minutes after a meal its flow is much diminished; at the end of this period it begins with increased rapidity, and so continues till the digestive process is nearly completed; and this increase occurred (though to a less extent) when no drink was taken with the meal.

The periods which elapsed between the taking of various substances into the stomach and their detection in the urine were various. Soluble saline substances passed, *cæteris paribus*, more rapidly than vegetable substances. The earliest period at which prussiate of potass was detected was about one minute after it was swallowed; the longest time that elapsed before it was found was thirty-nine minutes; and the chief source of this diversity appeared to be in the state of the stomach at the time. If the digestion of the last meal were finished, and the stomach empty when the solution of the salt was drunk, its passage to the urine was effected in one or two minutes; if the stomach were still digesting, its passage was retarded The time required for the passage of vegetable infusions was from sixteen to thirty-five minutes; it also varied, but in a less degree, according to the condition of the stomach.

Some other experiments were made to ascertain how soon after taking alkali-salts the urine would become alkaline. Solutions of the citrates and tartrates of potass and soda were given; and the time required for their decomposition and the appearance of their bases making the urine alkaline varied from twenty-eight to forty-seven minutes. In one case, after taking two drachms of bicarbonate of soda saturated with lemon juice, the urine did not till the sixth day recover its natural acid reaction. Its quantity was rapidly and greatly increased by taking the salts of soda.

* In a paper published in the Medical Times, April 4, 1846, from a Vienna Journal for March, Professor Hyrtl, maintains that the Malpighian capsules have no connexion at all with the urinary tubules, but open into the lymphatics. His account is said to have been drawn from most industrious, careful, and varied researches; but it is contradicted by observations which have been so often repeated and appear so secure from the danger of error, that I must for the present believe him to be thoroughly mistaken.

† Medical Gazette, June 27, and July 4, 1845.

[The publications on the chemical properties of the urine have been, in the last year, so numerous, that nothing less than a special report on them would give a sufficient account of their contents. I shall therefore only give the titles of the chief of them, and a brief notice of the nature of the contents of those which I have read. Many of them treat of things which are at present so far from being applicable in human physiology that the omission of a longer notice of them will not be regretted.]

E. v. Bibra. Ueber den Harn einiger Pflanzenfresser; in the Annalen der Chemie und Pharmacie, Januar, 1845; containing analyses of the urine of the horse, pig, ox, goat, and hare.

Golding Bird. On the mode of ascertaining the proportion of solid matters in the urine; in the Medical Gazette, May 23, 1845; giving a very useful table for ascertaining this at sight; a short rule being that if the sp. gr. of a sample of urine be expressed in four figures, (water being 1000,) the last two figures will nearly express the number of grains of solid matter in a fluid ounce of the urine; the quantity thus estimated being always rather less than the true quantity.

Boussingault. Recherches sur l'urine des animaux herbivores; in the Annales de Chimie et de Physique, Sept. 1845; analyses of the urine of the pig, horse, and cow, and an account of the relation which it bears to the food of each.

Chambert. Sur les sels et la densité des urines chez l'homme sain; in the Recueil des Mém. de Médecine militaire, t. lviii, and in the Annuaire de Chimie, 1846, p. 693. Data, but no definite conclusion, for estimating the relation between the sp. gr. of urine and its saline and organic contents; with new modes for certain parts of the analysis.

Heintz. Ueber die harnsauren Sedimente; in Müller's Archiv, 1845, p. 230; an explanation of the formation of the amorphons precipitates of urate of ammonia in cooling urine. The same chemist has published an essay on the mode of determining the proportions of urea, potash, and ammonia in urine, in the Annalen der Physik und Chemie, t. lvi, p. 114.

Bence Jones. On the variations in the alkaline and earthy phosphates in the healthy state, and on the alkalescence of the urine from fixed alkali; in the proceedings of the Royal Society, Jan. 19, 1845, and in the Philosophical Transactions, Part I, for 1845; showing the increase of the earthy phosphates after taking food, and their decrease after long fasting; also the increase of alkaline phosphates after feeding on bread alone, and after exercise; with other similar facts.

Laveran and Millon. Sur l'élimination de l'antimoine par les urines; in the Comptes Rendus, p. 237, t. xxi, and in the Annuaire de Chimie, 1846, p. 715; showing the intermittent and very slow elimination of the metal through the kidneys.

Marchand. On the composition of the urine of a tortoise; in the Journ. für prakt. Chemie, t. xxxiv, p. 244.

Möller. Ueber das Kystein; in Casper's Wochenschrift, Januar. 11, 18, 1845; a summary of his own and other's observations to show the little value of the presence of this principle in urine as a sign of pregnancy.

Pettenkofer. Ueber das Vorkommen einer grossen Menge Hippursäure im Menschenharne; in the Annalen der Chemie und Pharmacie, Oct. 1844. The urine of a girl thirteen years old, with chorea, contained 1·2886 per cent. of hippuric acid: a quantity equal to one fourth of all its solid constituents.

Pettenkofer. Ueber einen neuen stickstoffhaltigen Körper im Harne; in the Journal last quoted, p. 97.

Rabsky. A new mode of determining the proportion of urea; in the Annuaire de Chimie, 1846, p. 699.

Krukenberg. On the uniform influence of sweet fruit in making the urine alkaline, and the production of alkaline urine by the morbid secretions of the bladder after injuries of the spine; in the Oesterr. Medicin. Wochenschrift, 11 Januar. 1845, from Henle u. Pfeuffer's Zeitschrift, Bd. iii. [On all that relates to the progress of animal chemistry, or of chemistry in any other department, the reader may best consult the admirable Annuaire de Chimie of MM. Millon and Reiset. The Reports, also, by Dr. Day, in the 'Lancet' of February 1845, and in Dr. Ranking's Half-yearly Abstract, contain the substance of many of the best papers on animal chemistry.

### ORGANS AND FUNCTIONS OF ANIMAL LIFE.

*Structure of Joints.* Mr. Goodsir * points out the highly vascular fringes and processes of synovial membrane as more active in the formation of epithelium, and therefore more closely allied to the secreting organs, than other portions of these membranes are. The pulpy nature of their serous covering, their vascularity, and their position where they do not interfere with motion, but hang into the parts of the cavities which may be reservoirs of synovia,—all, he thinks, favour this opinion.

*Anatomy of the Knee-joint.* Dr. Gruber † has dissected 160 knee-joints to determine how, and how often, the bursæ near the joint communicate with it, and what are the usual arrangements of the recesses formed by the synovial membrane. The chief things he has noticed are—1. The sac which the membrane forms between the outer and posterior part of the joint and the tendon of the popliteus, and which covers the outer margin of the external semilunar cartilage, is often divided by a septum into two parts, placed one behind the other, and communicating by one opening with the joint. 2. The bursa under the quadriceps femoris is found in about every sixth or seventh person; and communicates with the cavity of the joint in about every ninth person. In 47 males it had a separate cavity in only 9; and in 33 females in only 3. 3d. The bursa between the tendon of the M. semimembranosus, the inner condyle, and the inner head of the M. gastrocnemius, is often locular or divided by septa into two or three cavities. In robust persons it is often, though not diseased, two and a half inches long and three fourths of an inch broad, so that it may be felt externally, and in them it generally communicates with the joint by an opening half an inch in diameter. 4. The bursa under the lig. patellæ was once found communicating with the joint; that under the inner head of the gastrocnemius never was: a communication between the femoro-tibial and tibio-fibular articular cavities was very rarely seen.

*Muscles of the Larynx.* Dr. Gruber ‡ has also found (but only once in a hundred dissections) a muscle which he names the M. thyroideus transversus anomalus. It is placed transversely over the upper two thirds of the middle crico-thyroid ligament, between the angle of the thyroid cartilage and the crico-thyroid muscles. Its greatest breadth is four and a half lines; its greatest width seven and a half. It has both its origin and insertion on the inferior angle and margins of the thyroid cartilage. The upper fibres are transverse and straight; the lower are longer and arched, with their convexities downwards. They are all fleshy at their attachments, and all have additional points of insertion on the crico-thyroid ligament. The action of the muscle is supposed to be assistant to the crico-thyroid muscles; to make the crico-thyroid ligament tense when the thyroid cartilage is fixed, and, when in full action, to approximate the alæ of the cartilage; or, when the cricoid cartilage is fixed it may draw the thyroid downwards and forwards.

* Anatomical and Pathological Observations, p. 42.
† Prager Vierteljahrschrift, 1845, B. i; and Oesterr. Med. Wochenschr., 25 Jänner, 1845.
‡ Oesterreich. Medic. Jahrbucher, Mai, 1845.

NERVOUS SYSTEM.

*General Structure, Origin, and Course of the Nerve-fibres.* The most important contribution to the physiology of the nervous system, perhaps, indeed, the most important physiological production of the year, is from Kölliker.* The main design of his essay is to discuss the question of the independence and speciality of the sympathetic nerve. This, it is known, has been long disputed; especially between Bidder and Volkmann on the one hand, and Valentin on the other. The former maintained, as the anatomical evidence of the independence of the sympathetic, that there belonged to it a peculiar set of nerve-fibres, characterised by their fineness, (they being only about half or one third as large as the cerebro-spinal fibres), their paleness, the absence of a double contour, their nearly uniform contents, and their yellowish gray colour when in bundles. The latter held that the sympathetic fibres are neither in structure nor in relations peculiar. The several statements on both sides have been nearly all inserted in former Reports.†

In the discussion of the question, Kölliker decides—1st. That the fibres described by Remak as peculiar to the sympathetic nerves, and which are commonly called Remak-fibres, are, as Valentin has always held, not nerve-fibres at all, but neurilemma, consisting of imperfectly developed fibro-cellular bundles. 2d. He determines that Bidder and Volkmann are right in their description of the structure of the fine nerve-fibres, or, at least, of the well-marked examples of them; and that these are not (as Valentin maintained that they were) Remak-fibres. But he denies that these fine nerve-fibres are peculiar to the sympathetic system, or even so different from the common larger cerebro-spinal nerve-fibres that they ought to be regarded as of a kind distinct from them. To justify this denial he shows that the characters assigned to these fine nerve-fibres as distinctive, by Bidder and Volkmann, are neither definitely marked, nor constant, nor essential; that there is no real difference between these fine fibres and those of the brain, spinal cord, and nerves of special sense; that, commonly, the large fibres assume near their peripheral ends the size and some other characters of the smaller ones; and that many fine fibres are found in all nerves, though it is generally true that there is a smaller proportion of them in the cerebro-spinal than in the sympathetic nerves.

But, although it thus appears to be an error to speak of sympathetic and of cerebro-spinal nerve-fibres as if they were two different *kinds* of fibres, yet the differences which do exist between them, and the various proportions in which the fine fibres occur in different nerves, make it important to discern their origin and course. On these points, Kölliker first proves the most important fact that these fine fibres have their origin not only in the ganglionic or nerve-corpuscles of the sympathetic ganglia, but in those also of the ganglia on the cerebral and spinal nerves, and in the corpuscles of the brain and spinal cord. In this, his observations fully confirm those of Helmholtz, Will,‡ and Hannover,§ who like him have seen this mode of origin, and of Bidder and Volkmann, who from another mode of investigation concluded that fine fibres must thus arise. Kölliker has seen this mode of origin of nerve-fibres in the spinal cord and in the spinal and sympathetic ganglia of the frog, in the spinal ganglia of the tortoise and cat, and in the Casserian ganglion of the cat and guinea-pig. Hannover has found it in all classes of vertebrata and in many invertebrata, in the brain and spinal cord, and in ganglia of all kinds; neither has he observed any other mode of origin besides this. The description given

* Die Selbständigkeit und Abhängigkeit des sympathischen Nervensystems; Zurich, 1844, 4to.

† Report on Microscopic Anatomy, pp. 33-5; Report for 1842-3, p. 18; Report for 1843-4, pp. 43-4-8. All that Bidder and Volkmann have maintained may also be found in the art. Nervenphysiologie, by the latter, in Wagner's Handworterbuch

‡ See last Report, p. 45.

§ Recherches Microsc. sur le Système Nerveux; Copenhague, 1844, 4to.

by Kölliker of the spinal ganglia of the frog is, that they contain one form of ganglion-, or nerve-corpuscles, which are of simple shape and give off no processes; and many other corpuscles, more or less pyriform, which at their smaller ends are drawn out into a process. This process, like the corpuscles, is pale and finely granular; it is from 1-10,000th to 1-7000th of an inch in diameter, and after proceeding about 1-1000th of an inch, it rather suddenly acquires a dark contour and slightly granular contents; it, in short, becomes a fine nerve-fibre. And, in regard to those cases in which he has not *seen* this mode of origin of the fine fibres, Kölliker so far confirms or admits the truth of Bidder and Volkmann's observations respecting the relative number of fine fibres which enter and leave the ganglia, that he considers it proved that a great number of these fibres have their origin in the ophthalmic ganglion, and in the ganglion of the vagus of fish; and considers it as highly probable that the ganglia of the cerebral and spinal nerves of all the higher animals are also sources of origin for similar fibres.*

To these observations may probably be added those of Dr. Todd and Mr. Bowman.† For although they do not demonstrate it, yet it is, as they state, most probable that one (or more?) of the processes of those which they name "caudate nerve-vesicles" is prolonged into a nerve-fibre. Their description of these vesicles or corpuscles is that they possess the general characters of the common nerve-vesicles, but have one or more long processes from their central parts or bodies. These processes, like the interior of the vesicle, are very delicate, frail, and finely granular. When unbroken, one or more of them may be traced, extending far from the vesicle, then dividing into two or three branches, which again divide, and give off extremely fine transparent fibres, such as may either connect distant nerve-vesicles, or become continuous with nerve-fibres. Such vesicles, they say, are best found in the locus niger, and in the gray matter of the cerebellum and spinal cord; and the tissue in their vicinity is freely traversed by delicate filaments which appear to be the ramifications of their caudate processes.

It has been already said that these fine fibres proceeding from the nerve-corpuscles are not peculiar to the sympathetic nerves (commonly so-called); yet all the branches of these sympathetic nerves contain a larger proportion of them than the common cerebro-spinal nerves do, and some of them contain no other fibres besides these. And assuming that the origin of these fibres is proved, the next question concerns their mode of distribution. Kölliker shows that there are much greater difficulties in the way of tracing these fine fibres from their origins than Bidder and Volkmann supposed. All that can be certainly said is that, 1st, the fibres arising in the sympathetic ganglia go partly to the viscera, and partly through their communicating branches, (which have been often called origins, or roots of the sympathetic, and are composed almost entirely of fine fibres,) to the anterior branches of the spinal nerves, in which most or all of them pass peripherally; 2d, some of the fine fibres which arise in the spinal ganglia pass through the communicating branches to the sympathetic, and are distributed in the viscera, and others go to the posterior branches of the spinal nerves; 3d, the fine fibres arising in the ganglia of cerebral nerves probably pass out from them with the nerves that are proceeding to their peripheral distribution. But it is yet uncertain whether the sympathetic ganglia‡ send fibres into the posterior branches of the spinal

* The most striking instance in which more fibres leave than enter the ganglia is seen in the septum of the auricles of frogs' hearts, which is so transparent that the ganglia and nerve-fibres may be counted in it. Here Bidder has often seen more fibres in one than in the other of the two branch from a ganglion—e. g. five in one, and seven in the other. Volkmann, in Art. Nervenphysiologie, l. c.

† Physiolog. Anatomy, p. 314, fig. 55-6.

‡ i. e. the ganglia of the sympathetic system commonly so called; the sympathetic system of Volkmann includes *all* the fine nerve-fibres, wherever they originate.

nerves; whether the spinal ganglia send fine fibres to their own anterior branches; whether the fine fibres arising in the spinal cord go to the sympathetic or to the spinal nerves, or to both; and what course the fine fibres which run with the spinal nerves take, though, from the large number of them in the branches of sensitive nerves, one may conclude that they are chiefly distributed with these.

As to the relative proportions of large and fine fibres in the nerves distributed to various parts, Kölliker concludes from his own and other observations that, 1st, the nerves of voluntary muscles contain in their trunks a majority of large fibres, but in their peripheral distribution either only, or a majority of, fine fibres; 2d, the nerves of the skin contain (for the most part) equal numbers of both; but in some of them, one or other size of fibres greatly preponderates, and in all of them the fine fibres greatly preponderate in their peripheral distribution; 3d, the nerves of sensitive mucous membrane are, in this respect, like those of the skin, except that in the nerves of the teeth-pulps and the gums there is a great majority of large fibres; 4th, in the nerves of involuntary muscles, and of the less sensitive or insensible mucous membranes, there is a great predominance of fine fibres.

To these important conclusions respecting the general anatomy of the nerve-fibres, a few less considerable facts may still be added.

*Structure of the Fibres.* Stadelmann* describes the axis-cylinder of the nerve-fibres as very distinctly visible in transverse sections of them. Its outline has commonly the same form, and is nearly half as large, as that of the nerve-fibre itself, but sometimes it looks like a mere chink or a central point.

Harting† has once noticed (but long after death) some peculiar fibres in the neurilemma of a nerve on the abdominal muscles of a Molge (Triton?) punctata. They were narrow, spirally twisted, band-like, with sharp outlines, and each of them had on its outer edge a row of very short ciliæ, which ended in minute round knobs, and were set at regular distances from one another. The average width of the fibres was about 1-1600th of an inch; the average length of the ciliæ 1-1100th; the diameter of their knobs 1-2300th; their distance apart about 1-800th.

*Central Terminations of Nerve-fibres.* Probably we must not conclude from the foregoing observations by Hannover, Kölliker, and others, that the fibres in the nerve-centres have no other mode of termination (if it may be so called) than that by connexion with the nerve-corpuscles. In the last Report, I mentioned the observations of Dr. Lonsdale,‡ who found, in two cases of anenaphalous monsters, that the nerve-fibres in the truncated portions of the fifth and other nerves, which hung unattached in the base of the skull, formed loops; a fact which seemed confirmatory of the theory of central terminal loops of the nerve-fibres in the brain. I have recently had occasion to confirm this fact in a mature fœtus, whose cerebro-spinal axis was truncated at the medulla oblongata. In the loose hanging ends of the fourth and fifth nerves, all the fibres appeared forming loops, exactly like those figured by Dr. Lonsdale. There can be no doubt therefore that this is the usual arrangement of the nerve-fibres in these cases, whatever may be the import of the fact.

*Peripheral Terminations of Nerve-fibres.* In doubt concerning the terminal loops of nerve-fibres, Volkmann§ tried the experiment of dividing half the

* Sectiones transversæ partium corporis humani, p. 17.

† Tijdschrift voor natuurl. Geschied. en Physiologie, 1845, d. xii, st. 1. In another examination he could not find these fibres. There is some similarity between this observation and that of Remak, in Müller's Archiv, 1841, p. 39, and Valentin's Repertorium for 1841, p. 108.

‡ In the Edinb. Med. and Surg. Journal, No. 157.

§ Wagner's Handworterbuch, art. Nervenphysiologie, p. 565.

trunk of the infra-orbital nerve, thinking that if there were terminal loops, at least several of the fibres divided might be connected at their peripheral parts with some of the undivided fibres; and that thus, on irritating the peripheral portion of the divided nerve, the impression might be conveyed centrifugally through its fibres, then through the loops and through the undivided fibres to which they led, and thus to the brain. But no pain was produced by the irritation; indicating either that sensitive nerves do not convey impressions towards the periphery, or else that there are no such peripheral loops as can convey impressions from one nerve-fibre to another.

*Termination of Nerve-fibres in the Pacinian Corpuscles.* The discoverer of the Pacinian corpuscles has continued his observations* on these bodies, and confirms the account of Henle and Kölliker recorded in the last Report. The nerve-filament within the corpuscle has, he says, a single contour, like the sympathetic filaments; up to the base of the corpuscle its contour is double. At its termination it presents a granular swelling like the common ganglion-corpuscle.

*Anatomy and Physiology of the Nervous Centres in general.* The researches of Hannover and Kölliker already reported, confirming and much extending those recorded in the last Report, render it necessary to admit the existence of many more nervous centres than are commonly reckoned. If under this title we may include all those bodies in which nerve-fibres have their origin or termination, and of which it can be made probable that they are centres or co-ordinators of the actions of many nerves, we must now include not only the brain and spinal cord, but, probably, all the ganglia on the spinal and cerebral nerves, and all those of the sympathetic system; for in all these we may believe, on the grounds already adduced, that there are nerve-corpuscles giving origin to nerves; and in many (as will presently appear), there is evidence of the same kind of action as in the acknowledged nervous centres. Not indeed that all these nervous centres have the same powers—they are not, like the brain, in direct communication with the mind, nor have all so wide a range of action as the cord; but each is a nervous centre to its own district, receiving from, and sending to, its own parts, the impressions which pass along the nerve-fibres which enter it. Each also is in communication with other centres, and, probably, through one or more of these, with the spinal cord; through which also, if not more directly, all may be connected with the brain, the head of the whole nervous system, through and beyond which no impression can be conveyed.

*Centres of the System of the Sympathetic Nerves.* The same researches of Kölliker and others show that this nerve, or system, may now be described as mainly composed, 1st, of ganglia, which (like other ganglia) contain (*a*) nerve-fibres traversing them; (*b*) nerve-fibres originating in them; (*c*) nerve-corpuscles giving origin to nerve-fibres; and (*d*) free nerve-corpuscles; and, 2dly, of various nerve-fibres, comprising (*a*) those which arise in their own ganglia; (*b*) those which it receives from the ganglia of the spinal and cerebral nerves; and (*c*) those which it receives from the brain or spinal cord, or both. Among the last are, probably, the large fibres, which are contained in some of the branches of the sympathetic, and through which the occasional influence of the brain and spinal cord upon the viscera is probably exercised.

Now all these same elementary structures occur in different proportions in the cerebro-spinal nervous system, and some of them pass from it into the sympathetic. Kölliker's conclusion is, therefore, that the sympathetic nerve is independent and peculiar, not by peculiar elements which are not found in

* Annali Univ. di Medicina; Luglio, 1845, p. 208.

other parts of the nervous system, but by its very numerous ganglia, by the fine fibres which proceed from their corpuscles, and by the general complexity of its composition; and that it is dependent on the other parts of the nervous system, in that it receives fine fibres from the ganglia of the cerebral and spinal nerves, and both fine and large fibres from the brain and spinal cord. Probably, however, it is in this respect more dependent in the higher than in the lower vertebrata.

And, as for the function of the sympathetic, these facts prove that its ganglia are so many centres of origin for some of its fibres; and they thus afford the anatomical evidence of what analogy and the physiology of the system had already rendered nearly certain, namely, that each ganglion of the sympathetic is, in its own sphere, a nervous centre, receiving, transmitting, reflecting, and, perhaps, even originating, the impressions on which the harmonious movements of the parts to which its nerves are sent depend. It is not necessary here to enumerate the organs thus dependent on nervous centres of the sympathetic, or the degrees in which they are severally subject to the influence of the brain and spinal cord. The best examples of movements governed by the sympathetic are afforded by the actions of portions of the heart, mentioned in a former part of this Report; by the many days' continuance of all the organic functions in frogs, after the removal of the brain and cord (saving the medulla oblongata);* and by the wide-extended influence of irritation of the intestines, if they are cut out with the mesentery (in which are their ganglia) still attached.

The influence of the sympathetic on the nutrition of the parts to which it is distributed is commonly known; such an influence appears to me to be constantly in exercise, affecting not only the quantity, but the mode, of nutrition in parts. I am surprised that so acute a physiologist as Kölliker should think this influence is exercised only in the power which it may have of determining the size of the blood-vessels; as if the vessels were not enlarged in many conditions, the results of which are all different; but I repeat what was stated in the last Report, that there is at present no evidence that the sympathetic system (i. e. the system commonly so called) exercises an influence on nutrition different either in degree or in kind from that which is exercised by the cerebro-spinal system.

*General Physiology of the Spinal Cord.* The general tendency of the investigations of the last year has been to prove that the spinal cord is neither a mere collection of tracts of nerve-fibres nor a single nervous centre, but (if I may use the most popular language of the day) a collection or series of *central stations,* each of which has its own lines of nerve-fibres terminating in it, and serves to receive, and to transmit on numerous lines and in various directions, the impressions which are conveyed by the centripetal nerves abutting on it. The chief evidence for this, which, though not a new view, has hitherto been a very doubtful one, is as follows:

1. Volkmann† has submitted the question whether the nerve-fibres of the spinal nerves remain and end in the cord, or go on to the brain, to the test of a kind of measurement. He weighed four pieces of a horse's spinal cord, each seven centimeters long, and taken respectively from below the 2d, the 8th, the 19th, and the 30th pairs of nerves. Their weights (in the order above named) were 219, 293, 163, and 281 grains; the areas of the transverse sections of the gray matter in them (in the same order) were 13, 28, 11, and 25 square lines; and those of the white matter 109, 142, 89, and 121 square lines. Thus, the quantity of white matter of the cord is absolutely less at the cervical

* See last Report, and Volkmann's Art. Nervenphysiologie, p. 500.

† Wagner's Handworterbuch der Physiologie, art. Nervenphysiologie.

than at the lowest part of the lumbar portion, and much less in the lower than in the upper cervical portion. The contrast was more marked in a comparison of the sum of the areas of transverse sections of all the spinal nerves of a serpent (Crotalus mutus) with that of a section of the upper part of the spinal cord. The former (purposely estimated below the truth) might be reckoned at ·0636 of a square inch; the latter only ·0058. The total size of the nerves, therefore, is at least eleven times greater than that of the cord—a difference which cannot be explained on the supposition that the nerve-fibres, when they pass into the cord, become smaller.

2. The almost necessary deduction from these facts is that many or all the nerve-fibres terminate in or very near those regions of the cord into which they penetrate; and this is strongly confirmed by the observations of Hannover and Kölliker, already often referred to, both of whom have demonstrated the fine nerve-fibres as prolongations of the processes of some of the nerve-corpuscles of the gray matter of the cord.

3. A step further is made by the remarkable observations of Volkmann, which have determined at least two examples of small portions of the cord having absolute and uninfluenced control over the movements of parts, to which parts they are the true and sole nervous centres. I refer here to the governance of the rhythmical movements of the lymphatic hearts by the two definite portions of the cord, of which an account has been already given. The evidence is complete that these portions of the cord are as truly the nervous centres for the two hearts as the portion of the medulla oblongata is for the respiratory movements.

4. Something of the same kind as this influence of the cord on the lymphatic hearts is indicated by an observation of Budge.* If a piece of the cord of a frog, scarcely two lines wide, be removed from the place at which the great brachial nerve goes off, it constantly occurs that the pulse of the heart decreases in frequency within two hours after the operation, and this does not happen when all the rest of the cord below this portion is removed.

But if it be thus proved that there are in the spinal cord many central stations, the question still remains, how an impression is conveyed from one to the other, or from any of them to the brain?† It is evident, that there are other modes of conveyance besides that through the continuous course of the fibres first impressed; it is not certain that any fibres pass uninterruptedly from the periphery to the brain, yet the impressions are precisely conveyed both to and from the brain; and there is no support in all these facts for the erroneous experiments of Van Deen,‡ which would have made it appear that not only the nerve-fibres, but the impressions also, stop short in or near the part of the cord on which they fall. Some of his more correct experiments show that even a small length of the gray matter left in the cord, when all around it is cut away, is sufficient for the conveyance of impressions up the cord of the frog, but how the conveyance is effected is as yet a question.

Some isolated facts concerning the anatomy and physiology of the spinal cord remain to be reported :—

Dr. Harless§ has described some singular results obtained by the action of a constant weak galvanic current on the spinal nervous system of frogs at the time of their greatest irritability, i. e. before the waking from hybernation. One pole was applied to the skin, the other to the spinal nerves of a beheaded frog. When the violent general convulsions had ceased, periodically inter-

* Oesterr. Medic. Wochenschr., 10 Jan. 1846 ; from Froriep's Notizen, 1845, No. 783.

† Hypotheses have been suggested in the year just passed by Drs. Todd and Volkmann (l. c.), but they both seem to me insufficient for the facts.

‡ See last Report, p. 50, and Report for 1842-3, p. 20.

§ Müller's Archiv, 1845, Heft i.

mittent convulsions, with regular intervals of rest from three to six seconds long ensued, and these often went on for one or two minutes. After these had ceased, there were no more spontaneous twitchings; but if, while the body was at rest, the slightest shock were given to the dish or the table on which it lay, it produced severe tetanic convulsions of the whole frame, which lasted from seven to eight seconds. The same convulsions were produced in an amputated leg subjected to the same influences; they were therefore not dependent on a reflex influence, but were direct. Careful examination showed that the shock which produced the convulsions did not at all displace the poles of the battery, so as to break and then renew the circle, and thus give each time a fresh galvanic irritation. And it was found that a shock, though slight, if given even to the connecting wire alone of the battery, was sufficient to excite the same tetanic convulsions. It was thus and by later experiments evident, that a shock communicated to a voltaic apparatus is sufficient to produce an alteration in the intensity of current, increasing it enough to produce convulsions in the animal galvanometer, and a greater deviation of the multiplier-needle.

*Cerebro-spinal Fluid.* M. Longet* has found that the peculiar unsteady tottering movements, like those of drunkenness, which M. Magendie ascribed to the removal of the subarachnoid fluid of the spinal cord, are really due to the division of the muscles of the occipito-atlantal region, which is made to form a passage through which the fluid may be drawn off. Whenever M. Longet drew off the fluid without injuring these muscles, the animal preserved the power of motion unimpaired; but when he divided the posterior sub-occipital muscles, (including always the recti capitis postici minores, and the supra-spinous ligament in the animals in which it exists,) the peculiar defects of motion were produced, although the cerebro-spinal fluid was left untouched, and the sheath of the cord unopened. He ascribes the impairment of motion in these cases to the falling of the head when its attachments to the atlas are destroyed, and the consequent dragging and pressure of the upper part of the cord, and especially of the medulla oblongata and pons. For the effects of the division of the muscles and other tissues are completely prevented by artificially supporting the animal's head in a raised position; and in different animals, the degree in which the movements are impaired is directly proportionate to the amount of separation which takes place between the occiput and atlas, when their connexions (the occipito-atlantal ligament excepted) are divided. The speedy recovery of the animal, which Magendie ascribed to the rapid reproduction of the fluid, M. Longet considers to be due to the readiness with which the nervous masses (especially in animals) adapt themselves to new and unnatural pressure. He observed a striking analogy between the effects of the division of these muscles, and those observed by M. Flourens and himself in consequence of injuries of the cerebellum; and hence draws another evidence, that the former are due to the pressure and dragging of the medulla and pons, with which the crura of the cerebellum are connected.

*Relation of the Nerve-roots to the Spinal Cord.* Van Deen† has published a plate from an accidentally-obtained preparation magnified 320 diameters, which appeared to show the mode in which the filaments of the nerve-roots are arranged in the substance of the cord. The central filaments of the root are drawn passing straight into the cord; the upper and lower ones passing in oblique lines upwards and downwards respectively. They all appear variously interlacing with the filaments of the cord, which, for the most part, run longi-

* Bull. de l'Acad. de Médecine, 15 Sept. 1845, and Gazette Médicale, 6 Sept.; and in other contemporary journals. See also Comptes Rendus, 7 Juillet, 1845.

† Tijdschrift voor Natuurl. Geschied. en Physiol., 1844, st. 2.

tudinally; but he could in no instance find a continuity between the two. His account thus far agrees nearly with that of Stitting.

Anatomy of the brain. *Cerebro-spinal Membranes and Vessels.* Among the observations which Purkinje* has republished from his Essay, first printed in 1838, are many concerning the nerves of the membranes and vessels of both the brain and the spinal cord. In the cerebral dura mater, the nerves are most abundant in the neighbourhood of the trunks of the three chief meningeal arteries. They come to these from the sympathetic system,† and the principal part lie in company with the arteries; but some also leave them and ramify separately in the substance of the membrane.

In the dura mater inclosing the spinal cord, Purkinje could not find a trace of nerves; but on the fibrous lining of the vertebral canal and the sinuses between it and the bodies of the vertebræ, (which fibrous lining may be regarded as an outer layer of the dura mater, divided at the foramen magnum,) he found abundant plexuses of sympathetic fibres.

The pia mater of the cerebellum displays many nerves, which branch separately from the arteries as those of the pia mater of the cord do; but they are less abundant than those are. The nerves ramifying on the pons and on the cerebrum appear, on the contrary, to belong exclusively to the arteries. In the choroid plexuses no trace of nerves could be found; but there is a dense plexus of filaments of the sympathetic system around the vena magna Galeni. It passes into the tentorium cerebelli, and appears to belong to it more than to the venous system.

In the pia mater of the spinal cord, a much more copious distribution of nerves exists than in any part of the cerebral membranes. The largest fasciculi, containing from thirty to fifty filaments, are near the anterior spinal artery, whence some pass into the process of pia mater in the anterior fissure, and form loops therein. Other large bundles which, for the most part, run longitudinally, are near the ligamentum dentatum, and about the posterior median line of the cord, though here they are less abundant than in front. In the neighbourhood of the origins of the spinal nerves, the bundles of sympathetic filaments are fewer and smaller. Their number is greater about the upper than about the lower part of the cord.

All the nerve-filaments of these plexuses in the pia mater appear to belong to the sympathetic system. They combine with those already mentioned on the pons and cerebellum; but have never appeared to be connected with the roots of the cerebro-spinal nerves. Some of the filaments come to the pia mater with its arteries; but they soon part company, and the nerves appear to increase, and form plexuses quite independent of the arteries.‡

*Dimensions of the Brain.* M. Baillarger§ has invented a new mode of measuring the surfaces of brains, by dissecting out all the white substance from their interior, and then unfolding the exterior, and taking a cast of it. From his measurements he estimates that the average superficial extent of the human brain is 669·3 square inches; and that it is far from true that, in general, the intellect of different animals is in direct proportion to their respective extents of cerebral surface. If their absolute extents of surface be taken, the rule is manifestly untrue in many instances; and it is not more

* Müller's Archiv, 1845, Nos. iii, iv.

† He suggests also that those which dissectors have supposed to be branches of the fourth and fifth nerves, going to the dura mater, are really branches of the sympathetic, which pass through these nerves to the membrane.

‡ As already stated, this account of the nerves of the membranes of the cord is confirmed and extended by the original observations of Mr. Rainey. It is also confirmed by Volkmann, in his article, Nervenphysiologie, already often referred to.

§ Gazette Médicale, 19 Avril, 1845; Report from the Acad. de Médecine, 15 Avril.

true if the extent of surface in proportion to the volume of the brain be regarded; for, according to M. Baillarger's measurements, the human brain has less superficial extent in proportion to its volume than that of [many] inferior mammalia.

Physiology of particular classes of nerves. *The Motor Nerves.* It has been already mentioned that E. H. Weber has successfully employed the continuous electric current developed by a rotating magnet for maintaining constant contraction of the voluntary muscles. He has also* applied it for testing the functions of the pneumogastric nerves; and it has been employed by Volkmann,† in an extensive series of experiments on the functions and modes of action of the motor nerves generally. The chief results are as follows:

1. The central organs, but not the nerves, are capable of an excitement which induces fixed muscular contraction even after the withdrawal of the external stimulus. For, with the magneto-electric stimulus applied directly to them or their nerves, the voluntary muscles, the œsophagus, separated fasciculi from the heart, and the iris (?) remain contracted just as long as the stimulus acts upon them, but no longer; but if the same stimulus be applied to the brain or spinal cord, the contraction of the voluntary muscles and œsophagus is prolonged after its withdrawal.

2. The motor nerves of the anterior, but not those of the posterior extremities [of the frog], arise from the uppermost part of the spinal cord. For if the anterior part of the cord be electrified, the contraction of the anterior extremities is often prolonged after the withdrawal of the stimulus; but that of the posterior extremities ceases with the cessation of the stimulus. Now, if the motor nerves of both extremities arose in the brain, this difference would be unaccountable; and as, according to the preceding rule, it is stimulus of the central organs alone which induces prolonged contractions, it follows that the upper part of the cord is a centre to the brachial, but not to the sciatic, plexus [of the frog].

3. The motor nerves of the heart, stomach, and intestines arise neither in the brain nor in the spinal cord; for none of these organs is thrown into fixed contraction when the brain and cord are directly stimulated by the magneto-electric current, although it is a law that all muscles will thus contract when either the origins or the trunks of their nerves are stimulated.

4. The heart has a central organ in itself. If, when cut out and whole, it is sufficiently excited by the magneto-electric current, it remains in fixed contraction after the current is interrupted; a condition which never happens in muscles separated from their centres, or in separated portions of the heart itself; for in these the contraction ceases with the stimulus.

5. Central organs modify the motor stimuli passing through them, and are thereby regulators of movements, [the *contractions* hitherto mentioned are states of fixed cramp-like contraction; the *movements* here named are movements of alternate contraction and relaxation]. For direct stimuli through the motor nerves always produce fixed contraction of their several muscles; but if the same motor nerves be acted on by reflected stimuli, then (even though these stimuli be continuously applied to the incident nerve, as by the magneto-electric current) *movements* of the muscles ensue, and these with order and an appearance of purpose. The replacement of the cramp-like contractions by the orderly movements in such a case, can be accounted for only by the intervention of the central organ through which the stimulus acts upon the motor nerves.

6. The heart, stomach, and intestinal canal possess central organs, a por-

* Archives de Physiologie, Janv. 1846. † Müller's Archiv, 1845, No. 5, p. 407.

tion of whose centripetal fibres lie in the vagus, the spinal cord, and the chain of the sympathetic. For when any of these parts of the nervous system is included in the magneto-electric current, there is no contraction of the heart, stomach, or intestines; whence it follows that neither their nerve-fibres nor their centres are directly stimulated in the experiment.* But orderly movements of alternate contraction and relaxation do, in this experiment, ensue in them; these, therefore, in accordance with the fifth rule, must be reflex movements, regulated by the nervous centres of the several organs, to which centres the stimuli were conveyed by fibres going to them from the vagus, sympathetic, or spinal cord.

PHYSIOLOGY OF PARTICULAR NERVES. *Third Nerve.* Among these experiments by Volkmann, are some† which show that when the third nerve is stimulated by the magneto-electric current, the voluntary muscles of the eye are thrown into contraction, which ceases on the instant of withdrawing the stimulus, while the contraction of the iris is sometimes prolonged beyond that time. In the voluntary muscles, the contraction indicates that they were stimulated directly through their nerves; in the iris it indicates a stimulus received by it through a centre; and Volkmann suggests that the difference is explicable only by supposing that there are in the trunk of the third nerve fibres which convey centripetal impressions to the ophthalmic ganglion, as to a centre from which the stimulus is conveyed to the nerves of the iris.

*Fifth Nerve.* Two interesting and well-observed cases of paralysis of the fifth nerve on the left side are recorded by Mr. Dixon.‡ In one (a woman fifty-three years old) there was (on the paralysed side) loss of motion in the muscles of mastication, and loss of common sensation in the conjunctiva, Schneiderian membrane, tongue, and skin of the face, except near and about the angle of the jaw. There was also complete loss of taste on the edge and forepart of the left half of the tongue, though on all other parts of the tongue the sense was perfect; and on the same side, there were loss of smell, partial loss of hearing, impaired vision, and cessation of the flow of tears while weeping at the other eye. Subsequently, the paralysis involved the third, and perhaps the optic nerve, of the same side. In the other case, the losses of nervous power were similar, but they were complicated by neuralgia of some of the branches of the fifth, and by inflammation (perhaps connected with the paralysis) of the conjunctiva, cornea, and other parts of the left eye. [In regard to the question whether the sense of taste be perceived through the fifth, or the glosso-pharyngeal nerve, these cases, like several others, are less decisive than at first they seem; for in those parts of the tongue in which the fifth nerve is distributed, it may only supply, as it does in the eye and the nose, a certain condition which is necessary in order that the special sense may be exercised through a special nerve. Even the facial nerve appears to have an office in the tongue essential to the sense of taste; yet it is not itself the nerve of taste].

M. Marchal (de Calvi)§ has related five cases, in which paralysis of the third nerve followed neuralgia of the fifth. One patient had diplopia without apparent deviation of the globe, which ceased on compression of the external frontal nerve. In two of the cases, the neuralgia followed a wound of a branch of the fifth nerve.

*Facial Nerve.* Under the title of the 'Anatomy of the Geniculate Ganglion,'

* In experiments by E. H. Weber (Arch. Gén. de Physiologie, Janv. 1846), it is said that the heart ceased to beat on strongly electrifying the pneumogastric nerves, and that its action was weakened and retarded when the stimulus of the nerves was less. † L. c. p. 426.

‡ Medico-Chirurgical Transactions, vol. xxviii, p. 373.

§ Bull. de l'Académie Royale de Médecine, 15 Oct. 1845.

Dr. Morganti* has published a long and important monograph on the facial nerve. He insists on the distinction of its two roots, reckoning as one the small fasciculus, the *portio intermedia* of Wrisberg. He traces, as Malacarne did, the principal root into the substance of the medulla oblongata, in which it at once divides into two fasciculi, one ascending, the other transverse. The first of these fasciculi is composed of longitudinal fibres, which seem to descend from the lateral tract of the medulla oblongata into the nerve; the second, or transverse, is composed of more numerous fibres, and looks like the continuation of the principal root of the nerve; it passes through the substance of the lateral tract, and is directed to its posterior surface, towards the median line of the floor of the fourth ventricle, where it meets its fellow of the opposite side, and is covered by the expanded gray substance of the cord.

The smaller root, or *portio intermedia*, may be traced to the external part of the posterior [restiform ?] tract of the medulla oblongata, in which it connects itself with the filaments of the vestibular branch of the auditory nerve, arising from the substance of the restiform body, where it combines to form the crus cerebelli.

By the aid of nitric acid and very careful dissection, Morganti has unravelled the communication between the trunk of the facial, this *portio intermedia*, and the vestibular division of the auditory nerve. The *portio intermedia* gives off a small branch, which unites with one from the vestibular, and in union with it enters the vestibular nerve. Then, next, it gives off two slender branches, which join the chief trunk of the facial, and cannot be traced further. Then an appearance of a plexus is produced by a branch being given off from this chief trunk, which branch crosses in front of the *portio intermedia*, and then winds behind it, looking just as if it were given to the *portio intermedia* and to the geniculate ganglion, but really passing them and rejoining the trunk. By separating this branch, it is shown that the geniculate ganglion is formed exclusively on the fibres of the *portio intermedia*, and has only a connexion of contiguity with the trunk of the facial. Further, it appears that the geniculate ganglion gives origin—1st, to the superficial petrosal nerves, (to the greater of which is added a small branch or two from the trunk of the facial); and, 2d, to two or three descending branches. These descending branches appear to join the knee-shaped bend of the trunk of the facial, but being unravelled they separate into—1st, numerous small branches forming a kind of plexus and then joining the main trunk of the facial; and, 2d, the chorda tympani, which the author holds, is thus formed from the *portio intermedia*, with the addition of one or two small branches from the trunk of the facial.

The author confirms this account by evidence from comparative anatomy; and he draws from the whole the conclusion that the geniculate ganglion (for the truly ganglionic nature of which he gives sufficient proofs) is to be classed with the ganglia on the posterior roots of the spinal nerves. In accordance with this view also, he holds that the facial is a mixed or double-rooted nerve, analogous to the fifth and spinal nerves; that the trunk (as it is here called) is its anterior root, and the *portio intermedia* its posterior root; that the superficial petrosal nerves are mixed branches, chiefly composed of fibres going from the facial to the spheno-palatine and otic ganglia; the chorda tympani, a mixed branch from the facial to the lingual branch of the fifth; and that, after the formation of the geniculate ganglion, the trunk and branches of the facial contain both sensitive and motor fibres continued from its roots.

The author opposes, in detail, all the objections to these views; and in answer to the belief that the trunk of the facial is insensible till it is joined by branches from the pneumogastric and fifth, and that the chorda tympani is

* Annali Universali di Medicina; Giugno, 1845.

composed of motor fibres exclusively, he adduces experiments. In many instances he divided the trunk of the facial directly after its exit from the stylo-mastoid foramen, (at least two inches further back than Panizza did); and in every one, acute pain was produced both by the division and by the irritation of the central portion of the trunk. The experiment was performed on horses, asses, dogs, and a sheep; and he shows reason for believing that the pain was not due to anastomoses of the fifth with the facial previous to its exit from the skull; though he admits that some of it might be due to the anastomosis of the auricular branch of the pneumogastric. In many other experiments he irritated the chorda tympani, (which he exposed through the posterior wall of the tympanum), and every time he did so the animal gave signs of pain.

M. C. Bernard* has added four cases of paralysis of the facial nerve, attended by impairment of taste, to those five cases which he published in 1843.† Dr. Guarini's experiments, showing that this is the motor nerve of the lingualis muscle, were noticed in the Report for 1842-3. M. Bernard‡ showed the same by experiments at nearly the same time as Dr. Guarini; and Dr. Verga§ corroborated them somewhat later: so that this fact may be considered certain. To prove the influence which the chorda tympani thus distributed in the lingualis muscle exercises upon the sense of taste, M. Bernard adduces these nine cases of disease of one of the facial nerves in men, and many experiments on animals. In all these, the taste of the corresponding side of the tongue was impaired; the flavour of quinine, citric acid, and other strong substances was very slowly and slightly perceived, while on the other side of the tongue the perception of them was instant and acute. He shows that this impairment is from the paralysis of the chorda tympani, by cases in which the cause of paralysis of the facial nerve being seated in front of the giving off of the chorda tympani, the taste was not impaired; and by experiments in which the facial being divided behind the part where the chorda tympani is given off, the sense was destroyed. By this last experiment, in which no injury could be done to any filaments of the Vidian that may join the facial, and by other experiments, which show|| that the chorda tympani is insensible, he makes it clear that the influence which the chorda tympani has on the sense of taste cannot be due to branches of the fifth nerve transmitted by the Vidian to the facial. Neither can the loss of taste be due to the dryness of the side of the tongue, for it is as moist as the other side. His own explanation is, that the papillæ of the tongue are rendered incapable of the vermiform movement by which they absorb the sapid particles, and bring them into contact with the gustatory nerve [a notion at least as improbable as any that his facts disprove].

In none of these four cases by M. Bernard was there any deviation of the uvula. They may, therefore, seem to confirm the evidence from Dr. Hein's¶ experiments that the facial does not send branches to the muscles of the palate or uvula.

But it is not certain that in these cases the seat of the disease of the nerve was behind the connexion of the petrosal nerves with it; and, if it were not, the muscles of the palate might be unaffected, though all the others supplied by the facial were paralysed. Notwithstanding these cases, therefore, and although Valentin** also has failed to excite movements of the palate in either the horse, dog, cat, or rabbit, by irritating the trunk of the facial nerve, yet, on the whole, I think the evidence is in favour of the opinion that, at least in man, the facial nerve sends filaments through the great superfical petrosal

* Archives Gén. de la Méd., Decembre, 1844.

† Annales Medico-physiologiques, Mai, 1843.

‡ In the Inaugural Thesis of M. Pomies, Paris, 1842.

§ Gazetta Medica di Milano, Giugnio 24, 1843.

|| According to Morganti, this is wrong; but the experiment is not essential to the conclusion.

¶ See last Report.

** Lehrbuch der Physiologie, ii, p. 673.

nerve to the spheno-palatine ganglion, and thence to the levator palati and levator uvulæ.* It is impossible otherwise to explain the deviation of the uvula, which is often seen when the facial nerve is paralysed. The absence of contraction of the palate-muscles when the nerve is irritated may be connected, as M. Longet suggests, with the filaments having to pass through a ganglion; in the same manner as irritation of the third nerve often fails to produce contraction of the iris.

*Pneumogastric Nerves.* Signor Poletti† shows that the removal of portions of both these nerves in the horse, sheep, and rabbit does not diminish the impression of the necessity of breathing. There is the same struggling for breath when the respiration is in any way hindered as there is while the nerves are entire; and if the animals, after division of the nerves, are put in an exhausted receiver, their anxiety for air is as great as when the nerves are not divided. In sheep, the glottis remains open though both the recurrent nerves are paralysed by the division of the pneumogastric nerves, and they breathe easily till the air-passages are obstructed by frothy fluid, or till the nostrils are purposely held close; then there ensues extreme dyspnœa. There were the same anxiety and dyspnœa when, after division of the pneumogastric nerves, the spinal cord in a rabbit was divided between the second and third cervical vertebræ; the diaphragm still acted with great energy. Poletti therefore supposes that, since in this last experiment nearly all the cerebro-spinal incident nerves were separated from the medulla oblongata, the sympathetic nerve must be an important agent in transmitting the impressions of the necessity of breathing.

From some experiments related by M. Dupuy‡ it would appear that in a few minutes after the ligature or division of the pneumogastric nerves, the blood of the carotid artery is black like venous blood, and contains less fibrine than it did before. If the life of the animal be prolonged for about six days by tracheotomy, the respiration becomes difficult, the signs of malignant pustule manifest themselves, and by placing portions of the spleen of such a horse in a wound made in a healthy horse, the disease may be communicated.

From many experiments on dogs, Dr. Mendelsohn§ deduces that the congestion and other disorders of the lungs which follow division of the trunk of the pneumogastric are due, not to the paralysis of the nerves of the lungs themselves, but to that of the branches of the recurrent on which the openness of the glottis depends; for the same affection ensues whether the recurrent or the pneumogastric trunks be divided. In both cases the glottis is nearly closed in inspiration, and the symptoms are the consequence of the diminished respiration. In regard to the diminished frequency of respiration after division of the pneumogastric, Dr. Mendelsohn says that the very same is observed after division of the recurrent nerves; and, in either case, may be prevented by the introduction of a tube into the trachea.

The continued irritation of this nerve by the magneto-electric current produces fixed cramp-like contraction of the œsophagus, and movements of alternate contraction and relaxation of the stomach. It follows, according to Volkmann's rules already mentioned, that the stimulus is conveyed to the œsophagus directly, through its motor fibres forming part of the trunk of the pneumogastric, and to the stomach indirectly by a reflected influence. Now, since the removal of the brain and spinal cord does not interfere with this

* In addition to the evidence for this view in the last Report, p. 52, see the cases concerning the diagnosis of fracture of the base of the skull in the Bull. de Thèrapeutique, Mars, 1845; and a case of tuberculous disease of the petrous bone, with paralysis of the facial nerve and deviation of the uvula, in the Oesterr. Medic. Wochenschr., 11 Oct., 1845.

† Bullettino delle Scienze Mediche, Luglio, 1844.

‡ Bull. de l'Acad. de Médecine, 31 Oct., 1844.

§ Roser and Wunderlich's Archiv, 1845, Heft ii; and Oesterr. Medic. Wochenschr. 19 Juli, 1845.

result, it follows that the stomach must have reflecting nervous centres in itself, and that there must be fibres in the pneumogastric nerves which are centripetal in their relation to these local centres.

*Accessory Nerve.* Volkmann* has repeated his experiments on the motor nerves of the larynx; and, in confirmation of his former statement and that of Dr. John Reid, says, that in numerous experiments he has never once seen movements of the muscles of the larynx follow irritation of the accessory nerves, but has as constantly seen them follow irritation of the pneumogastric. He has also completely divided all the roots of the accessory nerves in a dog, and has seen the movements of the larynx continue unchanged.

But after all the evidence adduced in the last two Reports in favour of the accessory being at least the motor nerve of some of the muscles of the larynx, I cannot think these experiments conclusive against it.

*Hypoglossal Nerve.* Dr. Stokes† has never, in many examinations, been able to find what Mayer described as the small posterior ganglionic root of the hypoglossal nerve in the ox. He has, however, found that it always has a small root derived from the side of the spinal cord anterior to the roots of the accessory nerve. From this origin the root passes up into the skull, and, without having any ganglion, is united to the lower of the two fasciculi in which the hypoglossal nerve passes through the dura mater.‡

### ANATOMY AND PHYSIOLOGY OF THE ORGANS OF SPECIAL SENSE.

OF THE EYE. *Vessels and Nerves of the Cornea.* Professor Gaddi,§ of Modena, describes, from a minute injection of the vessels of a child two years old, the vessels of the conjunctiva bulbi as forming a dense circle by numerous angular anastomoses around the border of the cornea. From the angles of union, he says, some very minute vessels are given off, which pass like rays, or like the vessels of the membrana pupillaris towards the centre of the cornea; and, besides these, there pass, from the same angles, many more, which insinuate themselves between the commissure of the cornea and sclerotica, and thus form communications with the vessels of the choroid.

Purkinje,‖ in the series of examinations already often quoted, has discerned, by making the cornea transparent in acetic acid, a "tolerably rich network of nerves" in it. These fibres variously combine with each other; those entering on one side from the ciliary nerves mingle with those from the other, so that no nerve-fibre appears to be lost in the substance of the cornea, or to pass into the superjacent conjunctiva.

*Structure and Movements of the Iris.* Some experiments of Signor Guarini¶ would make it probable not only that the movements of the iris are mainly due to two sets of muscular fibres,—one circular, for the contraction of the pupil, the

* Wagner's Handwörterbuch der Physiologie, art. Nervenphysiologie, p. 500.

† Cycl. of Anatomy, art. Ninth Pair of Nerves.

‡ Other recent works of interest on the physiology of the nervous system are—1. J. H. Lenzingar, Die menschliche Nervensystem physiol. bearbeitet; Zurich, 1845, 12mo—Idealistic. 2. K. F. Burdach, Umrisse einer Physiologie des Nervensystems; Leipzig, 1844, 8vo—Outlines. 3. Remak, Neurologische Erläuterungen; in Müller's Archiv, No. 5, 1844, containing, with controversial matter, brief accounts of small ganglia in the nerves in the heart, lungs, and larynx; of the six layers of the cortical substance of the brain; and of the central fasciculus in the nerve-tubules of the abdominal cord of the river cray-fish:—all according with former descriptions by the author. 4. Romberg, De paralysi Respiratoria; Berlin, 1845, containing the outlines of the physiology of the pneumogastric and its branches. 5. Cowan, Two cases of Carcinoma of the Brain, in the Provincial Med. and Surg. Journal, April 16, 1845. In one case loss of control over the muscles appeared to be owing to implication of the cerebellum: the patient, in walking, deviated to the left side, and the disease affected the left side of the cerebellum and pons, and the left crus cerebri. 6. Bourgery, Recherches sur le Système Nerveux Splanchnique; in the Gazette Médicale, 3, 10 Mai, 1846.

§ Bullettino delle Scienze Mediche, Nov. e Dicembre, 1844; Report from the Società Med. Chir. di Bologna, 30 Ottobre, 1844.

‖ Müller's Archiv, 1845, No. iv, p. 292.

¶ Annali Univ. di Medicina, 1844, and Gazette Médicale, 26 Avril, 1845.

other radiating, for its dilatation,—but, also, that these movements are under the influence of two sets of nervous fibres, of which one set, distributed to the circular muscle, are derived from the third nerve through the ophthalmic ganglion, and the other, distributed to the radiating fibres, are supplied by the branches of the cervical spinal nerves which pass through the superior cervical ganglion of the sympathetic. The experiments show, as Valentin's did, that after division of the third nerve the pupil dilates, and that after removal of the superior cervical ganglion it contracts. In animals killed by strychnine the pupil is dilated; but if, before giving the strychnine, one of the superior cervical ganglia be removed, and the pupil of the same side be thus made to contract, it dilates only a little after the administration of the strychnine, while, in the same case, the pupil of the opposite side is extremely dilated. Again, if one of the third nerves or the ophthalmic ganglion be irritated in a body still irritable, the pupil of the corresponding eye contracts slowly and does not dilate again. But if, after the pupil of a live animal has contracted on irritating the third nerve, the superior cervical ganglion be irritated, the pupil dilates again.

But while these movements of the iris after death and on the irritation of its nerves indicate its muscular structure, Guarini relates another experiment to show that the turgescence of its vessels has also some share in producing or maintaining the contraction of the pupil. Thus, he says that the contraction of the pupil, when after death the third nerve is irritated, is never so complete as it is during life; because the circulation having ceased, the congestion of the blood-vessels with which the muscular fibres are interlaced, and which they compress when they contract, cannot take place; but the dilatation of the pupil on irritating the superior cervical ganglion after death is as complete as during life.

*Membrana Pigmenti.* C. Bruch* describes the numerous round or oval nuclei among the pigment-cells scraped from the choroid, as forming a separate layer between the choroid and the cells of the pigment. Among those taken from the pig's eye, he found many fixed in a delicate, pellucid, structureless membrane; they lie, commonly, in close-set rows, or, more rarely, scattered in the membrane; and some, which have probably been accidentally separated, are found unattached. Bruch has traced this membrane in many animals, and in man, in whom it extends over the whole choroid, the ciliary ligament, and the posterior surface of the iris. Its nature and purpose are uncertain; whether it be only such a basement or germinal membrane as lies beneath the epithelium of mucous membrane in general, or a layer of embryo pigment-cells with cytoblastema, cannot be determined; but, if any part does, this deserves the name of membrana pigmenti.

*Tapetum.* Ernst. Brücke† has added observations to those which he published last year, to prove that the office of the tapetum is to reflect light, on the staff-shaped bodies of that part of the retina which is most used in vision, and so to make vision possible to certain animals, at times when those without a tapetum are in darkness. He has produced an extensive comparative anatomy of the tapetum; and has shown that while all the other various colours which the tapetum of the dog and other animals presents in different lights depend on it alone, the red colour which it occasionally reflects is due to the large vessels beneath it coming into view. And he has discovered that there are in different animals two distinct kinds of tapetum,—the one *fibrous*, the other *cellular*. The fibrous tapetum, which has been long known to consist of smooth, transparent, uudulating fibres, is found in ruminants, solidungula, the elephant, some marsupials, and the whale tribe. The cellular tapetum, hitherto unknown, is found in the carnivora and in seals. The cells, which may be best seen after pieces of tapetum have been macerated for a day or two in water with some hydrochloric acid or alcohol, are smooth, nucleated, elongated, unequally and imperfectly hexagonal, and very large, measuring from ·0008 to ·0028 of an inch in diameter.

* Untersuch. zur Kennt. des körnigen Pigments der Wirbelthiere; Zurich, 1844, 4to, p. 6.

† Muller's Archiv, 1845, Heft iv, p. 387.

With transmitted light they appear yellowish, and their nuclei pellucid; but with reflected light, they are beautifully blue and their nuclei black. They are peculiarly subject to deposits of earthy matter.

*Retina.* Pacini* has described the retina as consisting of five layers. The first or most internal is formed of the expansion of the white double-contoured (?) fibres of the optic nerve, lying close together in a transparent layer. The second is a single layer of nucleated and nucleolated transparent corpuscles, like simple ganglion-corpuscles, which he calls nerve-cells. The third, of a reddish-yellow colour, consists of gray nerve-fibres arranged in rays, which terminate in the cells of the second layer. The fourth is composed of several layers of corpuscles, identical with those described by Ehrenberg in the cortical substance of the brain and in other parts of the nervous system, and called by Pacini, from their nuclear characters, nuclear nerve-corpuscles. The fifth layer is formed by the little cylinders of the membrane of Jacob [the staff-shaped bodies of others]. Of these layers, the 1st, 4th, and 5th cease at the ciliary ligament; but the 2d and the 3d, as if forming together a true ganglionic system, appear to be continued over the ciliary processes and iris.

*Lens.* Sundry observations on the structure of the lens are published by Professor Harting.† Among them are the following. In both the new-born child and the adult he finds some of its flat fibres composed of rows of cells fixed end to end; and some having in or on their walls distinct round or oval nuclei, each with two or three very small irregular nucleoli. Both these kinds of fibres are found only at the equator of the lens, in which part they form the outermost layer. Neither the deeper layers at this part, nor the superficial layers near the poles, present a trace of such fibres. The lens of the new-born child has many more of the fibres with nuclei than that of the adult has.

In the lens of a cow, just beneath the surface, and in that of a titmouse (Parus major), near its nucleus, he found some of the fibres composed of from five to seven finer parallel fibrils; and in other fibres near the surface of the lens of the titmouse, there were transverse striæ, which (like Wagner, who has also observed such fibres) he compares to those of the primitive fibres of muscle.

*Vitreous Humour.* Ernst Brücke‡ has confirmed the account which he gave of the laminated structure of the vitreous humour by another mode of demonstration. If an eye, frozen hard, be slowly thawed, and the sclerotica and other membranes be separated gently as soon as they are soft enough, lamellæ of ice may be split off from the vitreous humour, exposing the layers of membrane of which it consists, the outer layers being parallel to the retina, the inner to the posterior surface of the lens; and all apparently structureless.

A more exact account of the structure of the vitreous humour is rendered by Hannover.§ He has examined it principally and with most advantage in the eyes of horses, hardened in chromic acid, so that sections in various directions could be made through them. By these means he has cleared up a doubt which Brücke's essay left, by finding that the several layers which compose the vitreous humour all form completely closed sacs, the outer sacs inclosing the inner, and all imitating the general form of the front of the eye behind the lens. Thus, in its laminated arrangement, the vitreous humour somewhat resembles a bulb, and the shape of its layers or sacs is such, that a line drawn from the middle of the optic nerve to the middle of the posterior wall of the lens would pass through the apices and the middles of the convex bases of them all.

On this same plan of concentric sacs of delicate membrane are formed the vitreous humours of the cat, dog, ox, sheep, and horse. But the human vitreous

* Atti della sesta Riunione degli Scienz. Italiani, in the Ann. Univ. di Medicina, Luglio, 1845.

† Tijdschrift voor Natuurl. Geschied. en Physiol., 1845, d. xii, st. 1. His account of the growth of the lens is mentioned under the head of Nutrition.

‡ Muller's Archiv, 1845, Hefte ii, iii. For his former account, see the last Report, p. 57.

§ Ibid. 1845, Heft v. There is in the same journal a paper by him on the arrangement of the fibres of the lens; but his description could not be understood without diagrams.

humour is differently constructed; it consists of sectors, all with their arcs directed outwards, and their angles converging to the axis of the eye. Its structure may be best compared with that of an orange, sections of which in various directions will display the various appearances which are seen in different sections of the human vitreous humour. In the axis of the eye, to which the angles of all the sectors of the vitreous humour converge, is the hyaloid canal, the passage which is open in the child for the arteria centralis retinæ; but the angles do not reach this canal; the substance next to it is uniform and textureless, as if all the sectors had become very thin, and had coalesced. The number of rays which may be seen diverging from this central part, and which represent the number of sectors, is about 180; the width of the arc of each sector is about one sixth of a line. It is not certain whether each sector has its own walls, or whether one wall serves for two. Whichever it be, these walls may be described as lamellæ of pellucid structureless membrane, extending from the tunica hyaloidea in rays towards the axis of the eye, and forming a kind of membranous skeleton for the more fluid part of the vitreous humour.*

*Relation of non-luminous Rays to the Eye.*—To determine why those rays which, after dispersion by the prism; are beyond the spectrum (the so-called chemical and calorific rays of light), do not produce on the eye the sensation of light, and to determine whether the retina is insensible to them, or they are hindered from reaching it, Ernst Brücke† has instituted some simple and very interesting experiments. The colour obtained from guaiacum wood is changed to a dark blueish-green on exposure to diffused light, the change being effected by the chemical rays,—those beyond and just within the violet. But if part of a surface tinged with the original colour be so screened that the light can pass to it only through an ox's or other animal's lens, the colour of this part is hardly changed. A part similarly screened, but having light admitted to it through a cornea, is only a little more changed; by light admitted through vitreous humour, yet a little more; and if to this screened part the light be made to pass through both a cornea and a lens, then, while all the rest of the surface undergoes the usual complete change of colour, this remains often unchanged, and is at most changed in only the slightest degree; so that it appears that the transparent parts of the eye absorb the chemical rays of light before they can reach the retina, and that therefore they are invisible.

Another set of experiments was directed to determine the relation of the calorific rays (those beyond and just within the red) to the same parts of the eye. These also appeared to be completely arrested; rays of light passed freely through the cornea and lens, and fell upon a thermo-electric apparatus; but its needle remained immoveable, as if the whole of the heating rays were absorbed.

EAR. *Movements of the Ossicula.* Eduard Weber‡ has found that the articulation of the head of the malleus with the incus is such, that when the malleus is moved with the membrana tympani, it cannot move upon the incus; but the two bones always move together, as if they were one bone. Their axis of movement is the line drawn from the slender process of the malleus to the lesser process of the incus, so that the membrana tympani impresses the movement on the malleus and incus, and these turn, as on a pivot, on the two processes adhering to the wall of the tympanum. Thus, when the membrana tympani is carried inwards, the stapes is pushed within the fenestra ovalis by the long process of the incus; and when the membrana tympani is carried outwards, the stapes is moved out from the fenestra ovalis.

* Hannover's attention was first directed to this structure of the human vitreous humour by a morbid examination of the appearance of its structure in two congenital cases of colobama, which are described in the same number of Muller's Archiv, p. 482.

† Muller's Archiv, Hefte ii, iii, 1845.

‡ Archives de Physiologie, Janvier, 1846; Report from the Naples Congress, 1845.

Hence appears the utility of the fenestra rotunda for the movements of the stapes. For the stapes could not thus move if the cavity of the internal ear was bounded by unyielding walls, since the fluid within it is hardly compressible. But as the fluid of the vestibule communicates with that of the cochlea, especially with that of the scala vestibuli, which again freely communicates with that of the scala tympani, so when the membrane of the fenestra ovalis is pushed inwards towards the vestibule, the membrane of the fenestra rotunda will be pushed outwards; as it is always seen to be after death in such a case. And thus the movements of the membrana tympani produce indirectly the flux and reflux of the fluid of the labyrinth, from the fenestra ovalis to the fenestra rotunda, by percussion and by the yielding of the lamina spinalis of the cochlea.

Tongue. *Structure of its Papillæ.* A new and very interesting account of the papillæ of the tongue is given by Dr. Todd and Mr. Bowman.* They have found that all those hitherto described as simple papillæ, appearing different by being variously grouped, are really compound organs; i. e. they are papillæ clothed with secondary, simple, and much more minute papillæ, which are concealed under the epithelial investment, and are scarcely or not at all visible till it is removed. They have also discovered minute simple papillæ, hitherto unknown, which are unequally dispersed among the compound ones, and occupy much of the space at the base of the tongue behind the circumvallate papillæ. The epithelium covering these makes the surface appear quite smooth; but when it is removed, they are seen prominent, each having an envelope of basement membrane, and receiving a capillary loop like those which pass into the simple papillæ of the skin. Papillæ like these compose the papillæ circumvallatæ. The surface of the fungiform papillæ is clothed with secondary simple papillæ, each of which receives a capillary loop from the more complex arrangement of vessels within the primary papillæ. The filiform and conical papillæ also are compound, being variously beset with fine acuminated secondary papillæ, more stiff and elastic than the others. The epithelium, also, of these papillæ (while that of the fungiform papillæ is very thin) is so thick and dense, that they appear white; it is also sent off from their surface in many fine hair-like processes, more or less stiff, or is arranged in variously imbricated scales, within which there are in some cases real minute hairs† pointed at the end, and having extremely fine central canals. Their structure sufficiently indicates that these papillæ are subservient to mastication rather than to taste.

### STRUCTURE AND FUNCTIONS OF THE ORGANS OF GENERATION.

*Nerves of the Male Genital Organs.* According to Purkinje,‡ fine-fibred nerves existed in the tunica albuginea in the neighbourhood of the epididymis. Many such, also, accompany the arteries of the penis. A rich network of large cerebro-spinal nerve-fibres exists in the fibrous tissue of the penis; and fine-fibred nerves with little ganglia may be found, but with difficulty, in its spongy tissue.

*Processus Vaginalis in the Female.* Professor H. Meyer § has described, and confirmed therein the accounts of Camper and some others of the older anatomists, the processus vaginalis of the peritoneum in the female fœtus. Its position and arrangement are exactly analogous to those which it has in the male.

*Maturation of Ova; Menstruation.* Two cases of apparent discharge of ova at the menstrual period are recorded by M. Serres.|| The ovum could not be seen in either, but in both, Graafian vesicles were found recently burst and very vascular.

* Physiological Anatomy and Physiology, vol. i, p. 435. The beautiful figures illustrating these structures describe them better than words can.

† The existence of these minute hairs guides at once to the explanation of the seemingly strange cases in which large tufts or coats of hair have been found on the tongue. See some such cases recently related by M. Landouzy in the Bull. de l'Acad. Roy. de Médecine, 15-30 Dec. 1845.

‡ Muller's Archiv, 1845, Heft iv, p. 293.

§ Ibid. Heft iv, p. 363.

|| Comptes Rendus, 18 Novembre, 1844.

Dr. Schweig* having ascertained in 60 women the length of interval between 500 occurrences of menstruation, finds that the mean length of the interval is 27·39 days; and that therefore the periodicity of menstruation corresponds, not with the phases of the moon, (for the lunar month marked by these is, on an average, 29·53 days); but with the nodes of the moon, the period from one of these to another of the same kind being 27·56† days.

Dr. Guy's‡ tables, made from observations of 1500 women, show that 15 years is the age at which the greatest number of women (English, I suppose) first menstruate; that the 14th year comes next in order, then the 16th, and that the 13th and 17th, 12th and 18th, 11th and 19th, present numbers which are successively less, but respectively nearly equal. Of the 1500, 791 first menstruated at 14, 15, or 16. Of 400 women, 159 ceased to menstruate between 45 and 50; 140 between 40 and 45; 51 between 35 and 40; 41 above 50; and 9 under 35. As a general rule, also, it appeared that the earlier menstruation begins the longer it lasts.

Mr. Roberton,§ continuing his researches into the question of the age at which women in different climates begin to menstruate, has obtained information from Moravian missionaries on the coast of Labrador, which proves that among the Esquimaux of that region the average period of first menstruation is the same as in this country. The fecundity of the Esquimaux women is also shown to be not less, but considerably greater, than that of the average of English married women; neither, since the introduction of Christianity among them, have early marriages been frequent.

He has also obtained tables‖ showing the average ages of puberty and first conception among Hindoo women. Among 71 at and near Bangalore, the average age at which menstruation first took place was 13 years and 2 months; the average age at the birth of the first child was 16 years and 5 months. Similar tables have been constructed for native women of Bengal, living in and near Calcutta, by Bábu Modusooden Gupta and Dwarkanath-das Bosu, the Demonstrator and Curator of the Calcutta Medical College.¶ In 152 women the average age at first menstruation was 12·48 years; two of these first menstruated when 8 years old; 2 when 9 years old; 2 when 17; 1 when 16. In 93 of these women the average age at which the first child was born was 14 years and 8 months; in 1 case only at 10; in 6 at 11 years; in 1 at 30; in 1 at 19; in 2 at 18. Yet the general result of the inquiries, though they show so early a period of puberty, is not wholly unfavorable to the opinion of Mr. Roberton, that the early puberty is the consequence of early sexual excitement; for girls are always given in marriage before their first menstruation, and it is customary to send them at as early an age as nine years to the houses of their husbands; and it is stated that if distance hinders this, menstruation is generally delayed till the 13th year. Some analogous instances of early fecundity brought on in animals by premature sexual intercourse are mentioned by Dr. Berthold.** Among them is one of a kid only fourteen days old which was impregnated by an adult goat; at the end of the usual period of gestation it bore a kid, mature, though very

* Gazette Médicale, 2 Août, 1845; from Roser and Wunderlich's Archiv für Phys. Heilkunde, Heft ii, 1845.

† So it is said in the original; but this period of 27·56 days is more nearly equal to the length of the *anomalistic* month of 27·5546 days, marked by the successive revolutions of the moon from perigee to perigee. The average interval between two menstrual periods, 27·39 days, is more nearly equal to the average length of the sidereal month, or 27·3216 days, in which the moon makes the complete circuit of the heavens. The true length of the nodical month is not 27·56 days, but 27·212 days. See Baily, Astronomical Tables.

‡ Medical Times, Aug. 9, 1845.

§ Edinburgh Med. and Surg. Journ., Jan. 1845.

‖ Ibid. October, 1845.

¶ India Journal of Med. and Phys. Science, February, 1845.

** Ueber das Gesetz der Schwangerschaftsdauer; Göttingen, 1844, p. 32. The accounts of the physiology of menstruation, by Raciborski and others, are examined by Dr. Alexander, in Oppenheim's Zeitschrift f. d. ges. Medicin, Dec. 1844.

weak, to which it gave milk, and which grew up healthy and strong. The young mother gave more than milk enough for its kid, grew up to full size and strength, and twice afterwards had kids.

*Processes following the Discharge of Ova; formation of Corpora Lutea.* Dr. Dubini* has carefully examined the ovaries in the bodies of many women, and has found—1. That in those who have never menstruated the ovaries have never shown any trace of cicatrices; but in those who have menstruated, cicatrices were never absent. 2. That in a few cases the ovaries of women who had been recently [but how recently ?] delivered presented no corpus luteum. 3. That the number of the so-called old corpora lutea, i. e. of vesicles with thick opaque white walls, and deeply wrinkled, which are found in the ovaries of different women, bears no relation to the number of children they have borne.

M. Raciborski† states that in regard to the formation of these bodies, which he supposes to consist in the "concentric hypertrophy" of the internal or granular membrane of the Graafian vesicle, there are characteristic differences between the females of the human species and of other mammalia. In the cow, ewe, doe, &c., the corpora lutea begin to be formed directly after the expulsion of ova, and they are just the same, whether the ova are impregnated or not; they are always genuine true corpora lutea. But in women, if the ovum expelled at any menstrual period is not impregnated, the *hypertrophy* of the internal layer of the vesicle is soon arrested, and it remains a thin yellow membrane in contact with the more or less altered clot of blood. If it is impregnated, the consequent process is different, but in degree only, not in kind; in this case the hypertrophy goes on, till very shortly the cavity of the vesicle is almost completely filled by the accumulated substance. And in this admission of the differences between the corpora lutea, according as the ova are impregnated or not, Berthold ‡ agrees; holding, I think, that the purpose of the development of the true corpus luteum is that a process may be going on in the ovary which may exclude the ordinary maturation and discharge of ova during gestation.

Some elucidation of what is thus roughly, though correctly, described as hypertrophy of the inner membrane of the Graafian vesicle, is afforded by the inaugural dissertation of Dr. Zwicky,§ who has minutely examined the formation of corpora lutea in cows and sows. The general results of his investigations may be thus briefly stated:—at the period of heat, when the ova are about to be discharged, the Graafian vesicle becomes more vascular and enlarges; serum is more abundantly secreted into it, and its membranes thicken. The cells also, part of which form the granular membrane of the Graafian vesicle, while part float in its fluid, are changed before the exit of the ovum. Flocculi appear in the fluid, and vascular folds and villi on the inner surface of the vesicle, which all consist of the altered cells. Some of the cells are changed by elongation into small narrow fibrous cells, which at last become fibres of imperfect fibro-cellular tissue; and others are changed into larger round or ovate cells, which also, unless they burst, are ultimately changed into similar fibres. And again, some other cells become four, or five, or even ten times as large as they were, and very finely granular; their nuclei also become larger and clearer, and their nucleoli more evident. Their enlargement appears to depend on the accumulation of the little fat-granules which the Graafian vesicles always contain. As they increase they pass from the round or ovate, into the oblong or acuminated form. The fat-granules and other fatty particles are those in which the yellow colour of the corpus luteum, when it exists, is contained.

When the ovum is discharged through the ruptured Graafian vesicle, blood is

* Annali Univ. di Medicina, Febbr., 1845, p. 277.

† Report from the Acad. des Sciences; Gazette Médicale, 23 Nov. 1844.

‡ Ueber das Gesetz des Schwangerschaftsdauer; Göttingen, 1844, p. 17.

§ De Coporum Luteorum origine atque transformatione; Turici, 1844, 8vo.

effused into the cavity, in the sow, but not, according to Zwicky, in the cow. The blood about half fills the enlarged cavity. After it has coagulated, it becomes gradually more solid, being compressed by the wall of the vesicle, which is both growing thicker and contracting. Thus compressed, and encroached on by the increasing and enlarging cells of the interior wall of the vesicle, the clot at last disappears, having, it would seem, contributed little to the formation of the corpus luteum.

The wall of the vesicle, after the exit of the ovum, is from half a line to two thirds of a line in thickness, and it continues to increase inwards by a continuance of the same process of multiplication and enlargement of its cells as preceded the discharge of the ovum, till, at length, the cavity of the follicle is completely filled. While it is thus increasing also, one portion of its fibrous cells are combined in fasciculi of connective or fibro-cellular tissue, which, traversing the whole mass, hold it together, and support the blood-vessels which run through it; and another portion are placed promiscuously between and among the large round cells. As soon as by this growth of the inner membrane of the Graafian vesicle its cavity is filled, its period of involution or degeneration begins; it grows smaller, more solid, and dries. In this change, some of its large round or ovate cells become narrow, and assume the appearance of fibres; and the longest, which are not thus changed, burst, and probably are absorbed with their contents.

The corpus luteum in this process of involution consists of only fibrous cells in various stages of development, and a large abundance of fatty matter. The fibrous cells become narrower, and their nuclei being drawn out, the whole gradually puts on the appearance of uniform slender cylindrical fibres, like those of immature fibro-cellular tissue. By these the walls of the Graafian vesicle are thickened, or else, as in cows, the fibres after a time coalesce with the stroma of the ovary, and can no longer be separated from it. And the end of the corpus luteum is, not that it is absorbed, but that it is lost sight of when this transformation of its component cells into fibrous tissue, like that of the stroma of the ovary, is complete.

*Gestation.* Dr. Berthold* estimates that parturition after healthy gestation takes place when the ovary, having omitted during pregnancy the maturation of nine menstrual ova, is preparing for the maturation and discharge of what, in the unimpregnated state, would have been the tenth. The proper period of gestation† will thus be equal to the interval comprised between the beginnings of ten menstruations, *minus* from ten to fourteen days. The above-named interval will not be always the same, while in different women, and in the same, under different circumstances, the intervals between the successive menstruations vary; but it may be estimated as equal to the number of days which intervened between the beginnings of the ten menstruations before the pregnancy. On an average this period will be between 290 and 300 days; the ordinary period of gestation will therefore be between 280 and 290 days; but it may be as short as 273, or as long as 291 days, in those whose habitual periods are shorter or longer than usual. In those whose menstrual periods are very short, parturition may take place at the eleventh instead of the tenth period.

Dr. Berthold's evidence for his mode of calculation is, 1st,—in a few cases in which it has proved true; 2d, in an apparent analogy in the case of cows and

* Ueber das Gesetz der Geschwangerschaftsdauer; Göttingen, 1844.

† This expression is not correct, because gestation is not begun till impregnation has taken place. The calculations are here made as if gestation began on the first day of a menstruation; but impregnation may take place some days (probably not less than twelve) after that day, or perhaps a short time before it. If, however, this mode of calculation is so correct, that the knowledge of the day of impregnation is unimportant, we must certainly admit Dr. Berthold's theory, that parturition is determined, not by the age or state of the child, or by the state of the uterus, but by that of the ovary, when it is reassuming its periodical action after its inaction of nine of its ordinary periods; a theory supported by the fact that, in pregnant animals, removal of the ovaries always produces abortion, but great injury of the tubes has no such effect.

sheep, in whom parturition occurs a little before the day on which the tenth heat-period would have fallen, if they had not been impregnated; 3d, in more distant analogies of some other periodic processes, such as the shedding of teeth and horns; 4th, the possibility of impregnation taking place in both women and animals within a few days after delivery; the ovum thus impregnated being probably that for the maturing of which the ovary was in preparation when parturition ensued; 5th, the apparently similar condition of the ovary shortly after delivery and shortly before the time of heat or menstruation.

*Lactation.* M. Dumas* has observed a constant difference between the milks of the truly carnivorous and the herbivorous animals, in that in the former the sugar of milk is either absent or exists in quantity too small to be extracted. But as soon as carnivorous animals (as bitches) are fed with bread or other amylaceous food, sugar is formed in the milk: and it disappears when their food is again made exclusively of animal principles. The casein of the milk of bitches is similar to that from the herbivora.

According to Dr. John Davy,† the coagulability by heat of the colostrum of the cow is due, not to its containing albumen, but to a peculiar modification of casein in it. Its nutritive matter is more concentrated, it is more easily coagulated by rennet, and it is less readily changed by the action of atmospheric air than the later milk is. The human colostrum (according to the examination of a few specimens) is not peculiarly rich in nutritive matter, neither does it coagulate on being heated.

* Report from the Acad. des Sciences, Gazette Médicale, 4 Oct. 1845; and in the Ann. des Sciences Naturelles, Sept. 1845.

† Medical Gazette, April, 18, 1845; and Medico-Chirurgical Transactions, vol. xxviii, 1845.

---

